# Assessing the effect of Loving-Kindness Meditation on pain and social well-being using a randomized controlled trial design

**DOI:** 10.1371/journal.pone.0357888

**Published:** 2026-09-16

**Authors:** Olivia Young, Katherine O’Connell, Naomi Nero, Melinda C. Somers, Paige A. Freeburg, Mary Ann Dutton, Abigail A. Marsh

**Affiliations:** 1 Department of Psychology, Georgetown University, Washington, District of Columbia, United States of America; 2 Interdisciplinary Program in Neuroscience, Georgetown University, Washington, District of Columbia, United States of America; 3 Department of Psychology, Yale University, New Haven, Connecticut, United States of America; 4 Department of Psychiatry, Georgetown University Medical Center, Washington, District of Columbia, United States of America; Universiti Sains Malaysia, MALAYSIA

## Abstract

Loving-Kindness Meditation (LKM), a contemplative practice that aims to promote social well-being, has also been linked to lower self-reported pain symptoms. However, it remains unknown whether LKM can reduce pain *through* having a positive impact on social well-being, as well as whether LKM can improve self-reported acute pain and objectively measured acute pain tolerance among a general, healthy population. In this study (ClinicalTrials.gov; NCT04632875), participants (*n* = 69) completed either a 4-week online LKM training or an active control meditation training (Progressive Muscle Relaxation; PMR), followed by a novel, remote pain induction task: the wall sit test administered over video conference. This study used a two-arm, parallel-group randomized controlled trial design with stratified block randomization and a double-blind baseline session. We hypothesized that, relative to the PMR active control, LKM would improve social well-being, reduce subjective pain reports, and improve acute pain tolerance. Contrary to our hypotheses, the PMR active control training increased perceived social support relative to LKM (*p* = .028), and neither meditation decreased subjective pain or increased acute pain tolerance over time. Our data were collected during the unique circumstance of the early COVID-19 pandemic, and it is important to interpret our results with consideration of widespread restrictions on in-person social contact and elevated stress during this period. This context may have constrained participants’ opportunities for social connection and influenced reports of social support and pain. Nevertheless, our findings add to the current understanding of how meditation, social wellness, and pain may be related.

## Introduction

Social connections are central to human well-being. Large, supportive social networks are associated with psychological wellness [1–3] and longevity [4–7], whereas a lack of social support is linked to deleterious health outcomes [4,7–9]. In particular, social well-being benefits physical pain through neural mechanisms such as the release of endogenous opioids from affiliative social behaviors [10,11], which can heighten pain threshold [12,13]. Conversely, social distress can exacerbate physical pain [14–17], poor social support is consistently linked to increases in reported pain [18–21], and individuals with fewer social relationships exhibit reduced pain tolerance [22].

One practice that may promote social well-being is Loving-Kindness Meditation (LKM) [23]. During LKM, meditators are instructed to cultivate feelings of kindness and love for themselves and then for a progressively distant series of others [24–27]. The explicitly social focus of LKM distinguishes it from mindfulness-based meditations, which guide participants to develop a neutral awareness of their present experience (e.g., by focusing on their breath) [28–30]. Prior research indicates that LKM improves well-being through elevated feelings of social connection [24,25,27] and heightened engagement in prosocial behaviors [26,31]. LKM also fosters positive emotions [24,32–34] and reduces physiological stress [33,35–37]—outcomes consistently linked to lowered pain in previous research [38–42]. A few intervention studies indicate that LKM may be able to directly reduce pain, including chronic lower back pain [43] and pain during and after breast biopsies [37,44]. However, although these studies suggest that LKM reduces self-reported pain in certain clinical populations, it remains unknown whether LKM training can improve acute pain among the general population. It is also unclear whether benefits to social well-being are the mechanism by which LKM affects pain, and whether LKM reduces both self-reported (“subjective”) pain and objective measures of acute pain tolerance. This study was designed to address these gaps in existing knowledge. Of note, this study was conducted during the early COVID-19 pandemic, a period in which many social relationships were ruptured, and is interpreted within this context.

**Fig 1:**
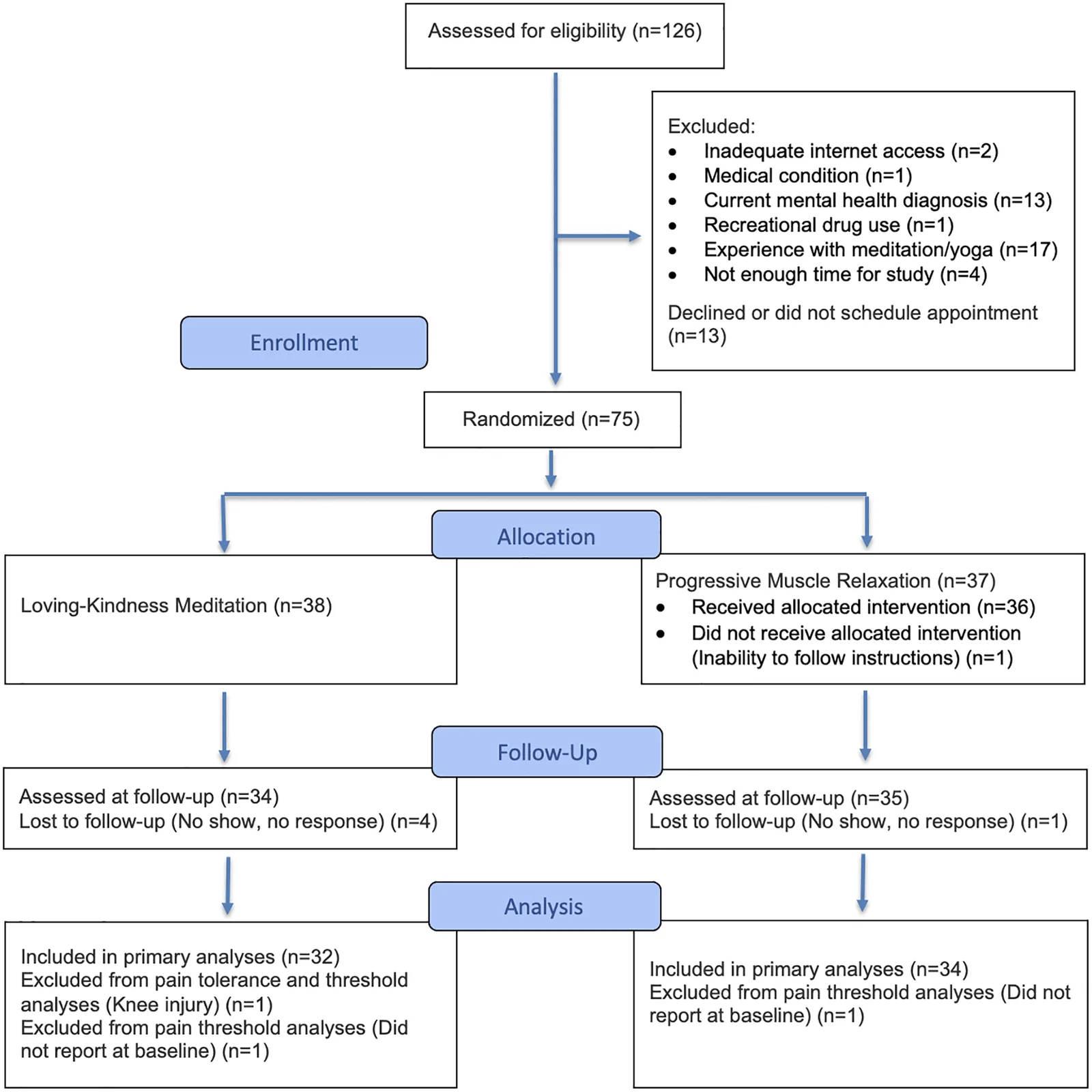
Consolidated standards of report trials (CONSORT) flow diagram. Participants who were assessed for study eligibility, allocated to randomized intervention conditions, lost to follow-up, and included in the finalized analysis.

In a two-arm, parallel-group randomized controlled trial that allocated 75 adults (Fig 1), we compared the effects of LKM to Progressive Muscle Relaxation (PMR), a meditation practice that is known to reduce anxiety [45–47] but has not been found to improve feelings of social connection like LKM [48]. We assessed objective measures of pain (pain tolerance and pain threshold) and subjective ratings of pain remotely by administering a wall sit test [22,49–52] over video conference. PMR was selected as the active control because it is matched to LKM on several features: both interventions could be delivered as 15-minute audio meditations, both cultivate a directed attentional focus, and both have plausible relaxation-related effects. In addition, because PMR has decades of validated use for stress and anxiety [53], control-assigned participants would be unlikely to consider the program inert, and less likely to drop out. PMR interventions have shown beneficial effects on fatigue and self-reported pain in prior research with clinical populations experiencing chronic pain [54,55]. They could reasonably also improve pain among non-clinical populations, potentially through a physiological relaxation mechanism. Critically, however, PMR lacks any explicitly social, compassion-oriented content that is the theorized active ingredient of LKM. Because PMR serves as a true active control, the design and assessment of social changes in this study were important to ensure we could capture and accurately attribute potential impacts on pain to the hypothesized, social pathway of change.

**Fig 2:**
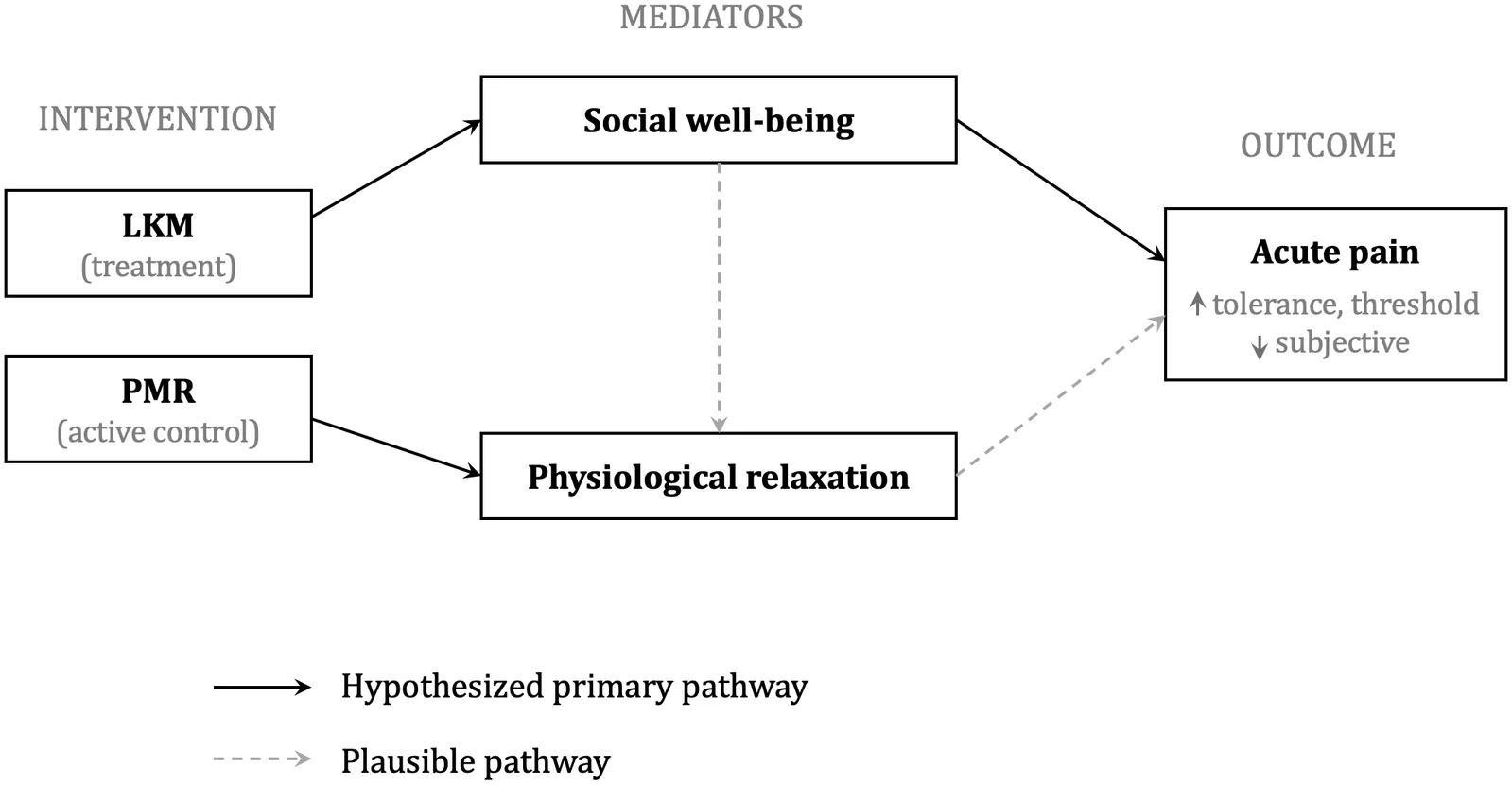
Conceptual model. Hypothesized pathway by which Loving-Kindness Meditation (LKM) may increase acute pain tolerance and reduce subjective pain through improvements in social well-being (social support, loneliness, prosocial and social activity). Progressive Muscle Relaxation (PMR) serves as an active control matched on format, dose, attention, and relaxation, and is not hypothesized to affect pain through a social pathway.

We also evaluated both subjective and objective components of social well-being. Subjective measures of social well-being included self-reported loneliness, anxiety, and social support, whereas objective measures included assessments of engagement in helping behaviors and other social activities. To account for COVID-19-specific social factors, we measured local rates of COVID-19 cases, deaths, and vaccinations, as well as self-reported social distancing, face mask use, emotional isolation, and physical isolation. We hypothesized that LKM would increase acute pain tolerance and threshold, and reduce subjective pain reports, with the beneficial influence of LKM on social well-being as a mediator of these effects. Fig 2 summarizes this hypothesized pathway, in which LKM enhances social well-being, which in turn raises acute pain tolerance and reduces subjective pain. Since improved social well-being could also increase physiological relaxation, which could potentially impact pain on the wall sit test, the figure includes a plausible secondary pathway by which LKM improves acute pain through relaxation. This secondary pathway was not hypothesized, and, similarly, we did not expect PMR to impact pain, but these potential relationships are included in Fig 2 to recognize them as possible.

## Materials and methods

The present study was conducted between December 2020 and July 2021 using email communications, phone calls, online video conference (Zoom.us), and Qualtrics Survey Software. All study procedures were carried out in accordance with a protocol that received Institutional Review Board approval from the Georgetown University Institutional Review Board. Prior to testing, all participants provided written informed consent via DocuSign. The study protocol was registered as a clinical trial on ClinicalTrials.gov on November 17, 2020 (NCT04632875; see supporting information) and the hypotheses, study design, and planned statistical analyses were preregistered on Open Science Framework on February 19, 2021 (https://osf.io/hz3nv).

### Sample size

When determining sample size, we aimed to test for a treatment effect size of partial eta-squared n_p_^2^ = 0.02 for change in pain tolerance, as detailed in our preregistration. This target effect size was selected as a conservative estimate consistent with the modest effects reported in prior research linking social interventions and acute pain in healthy adults [22,41]. An a priori power analysis conducted in G*Power indicated that a total sample of N = 68 (n = 34 per arm) would provide 90% power to detect this effect in a 2 (meditation group) × 2 (time) repeated-measures design at a two-tailed α = .05, assuming a correlation between repeated measures of r = .75. With this sample size we additionally expected greater than 80% power to detect group differences in self-reported social well-being outcomes, based on expected effect sizes of Cohen’s d = 0.83–1.45 reported in prior LKM research [27,56], using a Bonferroni-adjusted α = .05/4 = .0125 to allow for four planned comparisons. To accommodate an anticipated attrition and non-response rate of approximately 10%, target enrollment was set at 74 participants. Although a priori sample size calculations used a power-based approach, we expected reasonable precision around our estimate of the treatment effect on change in pain tolerance.

### Participants

Healthy adults were recruited using ResearchMatch, ClinicalTrials.gov, online advertising, electronically-distributed flyers, and word of mouth. Participants were required to reside within the United States, which was confirmed using geolocation data from Qualtrics. An initial phone screening was used to determine if participants were eligible to enroll in the study. Participants were excluded if they reported a current mental health disorder (in response to an initial question: “Do you have a current diagnosis of a mental health disorder?” and a later, confirmatory question: “Do you currently have any diagnosed psychiatric conditions?”), current use of any psychoactive medications, current use of illegal drugs, current use of recreational drugs (with the exception of infrequent (≤1x/month) marijuana use), any chronic pain condition (including, but not limited to any low-back pain, joint pain, migraines (>1x/month), arthritis, or idiopathic pain), current or past neurological disorders, current regular use of pain-relieving drugs, being pregnant or planning to become pregnant in the near future (added to exclusion criteria after preregistration for the purpose of participant safety), or any contraindications to safe participation (e.g., knee injuries that could worsen during the wall sit test, vasovagal syncope, or a history of fainting episodes during indoor exercises like the wall sit test). Participants who reported a lack of internet access, previous formal meditation training (≥3 sessions), current practice of meditation formally or informally, or current frequent practice of yoga or other similar mind-body practices (≥1x/week) were also excluded. All participants were between the ages of 18–55 and reported interest in trying meditation. Short descriptions of the LKM and PMR practices were provided during the phone screening and eligible participants were aware they would be randomly assigned to one type of meditation after the baseline session. All participants reported having internet access, the ability to join an online Zoom session with video, and the ability to complete online surveys on either a computer or a smartphone. Numbers of participants who were enrolled, randomized, and included in final analyses can be seen in Fig 1.

Seventy-five adults were enrolled in the study, and the final sample included 69 participants, which met the pre-registered target (Table 1). Six participants did not complete the study due to an inability either to follow instructions in the baseline session (*n* = 1) or to be contacted for follow-up (*n* = 5; three females, one male assigned to LKM, and one female assigned to PMR; they completed 13, 12, 0, 0 and 1 meditation sessions, respectively). One participant did not complete the wall sit test at the follow-up session due to a recent injury but was retained in the social well-being analyses and final sample. Two participants forgot to indicate pain onset during the baseline session and therefore were not included in the analyses of pain threshold. Participants were compensated $90.00 for completing the study (approximately $10/hr).

**Table 1:** Sociodemographic Characteristics of Participants at Baseline.

	LKM	PMR	p-value ^a^
N	34	35	
Age ^b^	30.2 (8.9)	31.7 (9.2)	.518
Gender			.873
Female	22 (65%)	22 (63%)	
Male	12 (35%)	13 (37%)	
Other	0 (0%)	0 (0%)	
Race ^c^			.459
White	19 (58%)	24 (69%)	
Asian	8 (24%)	7 (20%)	
Black/African American	2 (6%)	3 (9%)	
Other/Multi	4 (12%)	1 (3%)	
Education			.076
High School Graduate or G.E.D.	0 (0%)	1 (3%)	
Some College	7 (21%)	2 (6%)	
Two-Year College Degree (A.A.)	2 (6%)	3 (9%)	
Four-Year College Degree (B.A., B.S.)	16 (47%)	11 (31%)	
Master’s Degree	9 (26%)	14 (40%)	
Doctoral or Professional Degree	0 (0%)	4 (11%)	
Average daily physical exercise			.370
Not at all	0 (0%)	1 (3%)	
< 30 min/ day	19 (56%)	19 (54%)	
30–59 min/day	14 (41%)	11 (31%)	
60–90 min/day	1 (3%)	4 (11%)	
Local Covid-19 cases per 100k ^b^	23.2 (14.3)	29.2 (24.6)	.223
Social Support (ISEL) ^b^	40.5 (6.8)	40.4 (4.8)	.944
Loneliness (UCLA v3) ^b^	39.3 (12.8)	37.6 (8.5)	.505

^a^p-values were obtained with a two-tailed t-test for continuous variables and a chi-squared test for categorical variables.

^b^Values are presented as mean (standard deviation).

^c^One participant in the LKM group did not report race.

### Study design

We employed a two-arm, parallel-group randomized controlled trial design with a 1:1 allocation ratio comparing LKM to an active PMR control. Participants completed one baseline and one follow-up testing session remotely through online video conference (Fig 2). Both the baseline and follow-up sessions included acute pain testing using the wall sit test and questionnaires assessing subjective social well-being. Online testing sessions also included an experimental pain test that involved holding an ice cube and a short social reinforcement learning task. These two tasks were always administered last and are not discussed here. Upon completing the baseline session, participants were randomly assigned to either four weeks of LKM or four weeks of PMR. Random assignment used block stratified randomization to ensure equal allocation by gender and age group (<30 years or ≥30 years of age). The researchers were unaware of participant assignment until after baseline testing and used a sequential opening of envelopes organized by stratification to remain blinded until then.

After random assignment, participants completed five consecutive days of daily experience sampling to assess objective social well-being and COVID-19-related variables. Within seven days of completing the daily experience sampling, participants began the online LKM or PMR training. The meditation training program involved six 15-minute meditation sessions per week for four weeks, totaling 360 minutes of LKM or PMR across 24 sessions. Each session involved listening to a pre-recorded guided meditation audio file through Qualtrics, which enabled real-time tracking of each session’s duration and completion status. Online administration of meditation provides several advantages: 1) it was safer than face-to-face meetings during the ongoing COVID-19 pandemic; 2) participants often prefer online meditation training over individualized or group face-to-face training [57]; 3) it overcame common barriers to treatment by combining easy access, low cost, and a degree of privacy; 4) it ensured that social changes were not influenced by direct exposure to other people.

**Fig 3:**
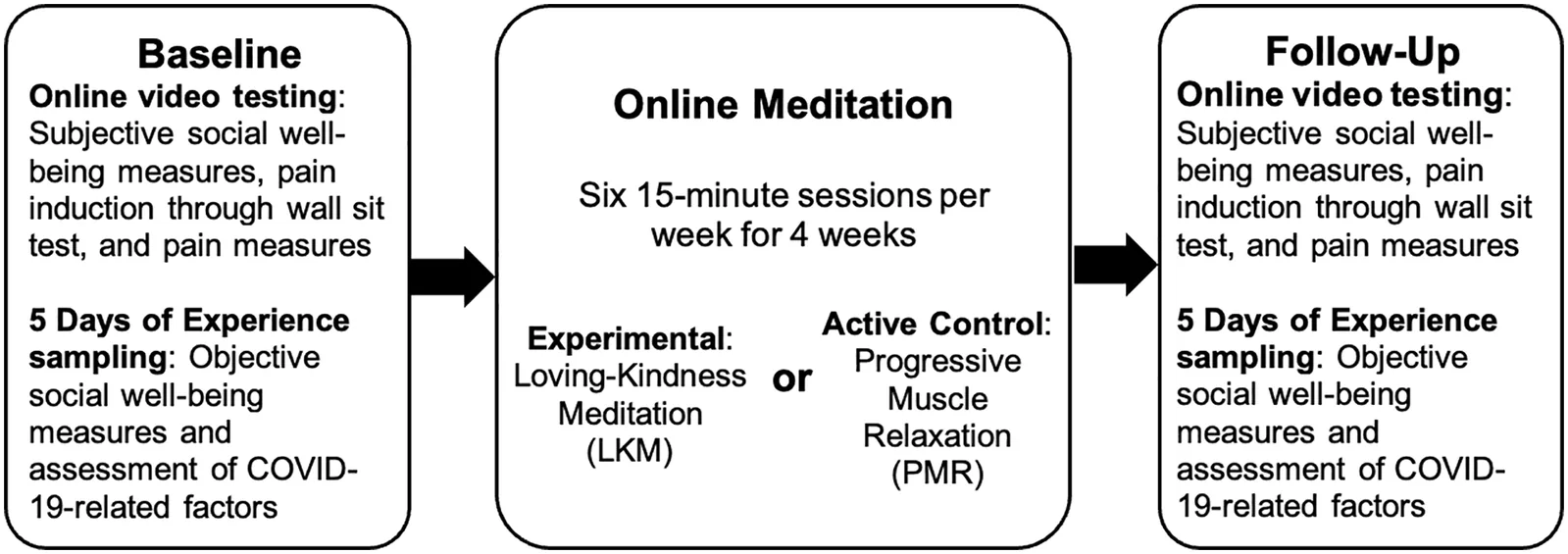
Outline of study design. Eligible participants completed a baseline testing session on online video conference followed by five consecutive days of daily experience sampling. Participants then completed four weeks of meditation (randomized to either LKM or PMR) followed by a second testing session on online video conference and an additional five days of daily experience sampling. The average time between remote baseline and follow-up sessions was 36.7 days. LKM = Loving-Kindness Meditation; PMR = Progressive Muscle Relaxation.

Participants were instructed to complete the meditations in a quiet environment at home. Meditation sessions were only counted as completed if the duration of the meditation had elapsed and the participant confirmed they had listened. Emails containing the link to the meditation were sent to participants six days each week. Participants chose in advance the time of day they preferred to receive the emails and which six days of the week they preferred to complete the meditations. If participants missed or only partially completed three days of training within a five-day period, they were emailed a reminder and given the opportunity to make up any missed sessions. Within seven days of completing their final meditation session, participants were scheduled to complete the follow-up session. The follow-up session included the same wall sit test, pain measures, and subjective social well-being measures as the baseline session, as well as an additional brief interview at the end. After the follow-up session, participants completed another five consecutive days of daily experience sampling to measure objective social well-being and COVID-19-related variables (see Fig 3). Participants were paid one sum after completion of the full study or at the time of withdrawal. Due to the low-risk nature of the study, harms were not systematically assessed and we did not employ stopping guidelines.

### Description of the intervention

#### Loving-Kindness Meditation (LKM).

The goal of LKM is to generate universal feelings of kindness and compassion towards others [23]. World-renowned LKM expert Sharon Salzberg [23], who has more than 40 years of experience teaching meditation, created a LKM meditation program for our research group and led each pre-recorded LKM audio meditation administered in this study. A 12-minute introduction to LKM was included prior to the first meditation session, which explained the goals of the practice and provided practical recommendations for novice meditators (e.g., to sit or lay down comfortably and to keep eyes closed). Each subsequent meditation was 15 minutes long, and every three sessions, the focus of the meditation changed to a new person or group of people (with eight different foci across 24 meditations). LKM sessions were aimed at progressively expanding feelings of closeness, in the following order: toward (1) the self, (2) a benefactor, (3) a neutral person, (4) a friend, (5) a distant friend, (6) a difficult person, (7) a group of individuals, and (8) all beings. After generating a clear image of the person(s) of focus, participants were guided to offer them wishes of loving-kindness for well-being, safety, happiness, health, and peace. No background music was included.

#### Progressive Muscle Relaxation (PMR).

An active control training consisted of four weeks of online Progressive Muscle Relaxation (PMR), which has been used in previous studies for comparison with LKM due to its non-social focus [48,58]. Like LKM, each PMR meditation session involved listening to a 15-minute pre-recorded guided audio meditation, six sessions were provided per week, and every three sessions introduced a new focus on different parts of the body. A four-minute PMR introduction was included prior to the first session, which explained the goals of the practice and provided practical recommendations for new meditators. PMR sessions were aimed at progressively expanding focus on different parts of the body: (1) hands and arms, (2) face, (3) head, neck and shoulders, (4) abdomen and buttocks, (5) legs and feet, (6) all upper body, (7) all lower body, and (8) whole body. The PMR scripts were adapted from a commercial program and read by a female professional guided meditation narrator. No background music was included, and the PMR recordings are publicly available on Open Science Framework.

### Subjective social well-being measures

#### Social support (ISEL-12).

The 12-item Interpersonal Social Evaluation List (ISEL-12) assesses perceived quality of interpersonal support [59]. The ISEL-12 includes the four highest loading component items from three subscales in the original 40-item ISEL: Appraisal (e.g., “When I need suggestions on how to deal with a personal problem, I know someone I can turn to”), Belonging (e.g., “If I wanted to have lunch with someone, I could easily find someone to join me”), and Tangible (e.g., “If I were sick, I could easily find someone to help me with my daily chores”). Items are scored on a four-point scale (0 = “Definitely False” to 3 = “Definitely True”), with six items being reverse scored. ISEL-12 total scores, which range from 0–36, show excellent reliability (Cronbach’s alpha = 0.80–0.90) [60,61].

#### Loneliness (UCLA v3).

The 20-item UCLA Loneliness Scale-Version 3 (UCLA v3) assesses feelings of loneliness [62]. Example items include: “How often do you feel that you lack companionship?”, “How often do you feel that your relationships with others are not meaningful?”, and “How often do you feel close to people?” (reverse scored)*.* Items are scored on a four-point scale describing the frequency of feelings (1 = “Never” to 4 = “Always”), with nine items being reverse scored. UCLA v3 total scores, which range from 20−80, show high reliability (Cronbach’s alpha = 0.89–0.94) [62].

#### Anxiety (STAI-Y2).

Participants completed the revised Form Y2 of the Spielberger State-Trait Anxiety Inventory (STAI-Y2) [63,64]. The STAI-Y2 is a 20-item scale that measures how anxious respondents typically feel (i.e., trait anxiety) with example items that include: “I feel nervous and restless”, “I lack self confidence”, and “I make decisions easily” (reverse scored). Items are scored on a 4-point scale from 1 = “Almost never” to 4 = “Almost always”, with nine items being reverse scored. STAI-Y2 total scores, which range from 20–80, demonstrate excellent reliability (Cronbach’s alpha = 0.91–0.93) [64,65]. Notably, recent work shows low discriminant validity of the STAI-Y2, indicating the scale may actually measure negative affect more broadly because scores are highly positively correlated with both depression and anxiety [66].

#### Objective social well-being measures.

Daily online surveys were sent to participants at 7:00 pm local time via Qualtrics for five consecutive days before starting LKM or PMR and again shortly after completion. The five days always included three weekdays and two weekend days. Participants were instructed to complete the survey before going to bed at night and to answer questions related to their behavior that day [67]. The survey was adapted from validated checklists of prosocial behavior [67,68] and social activity [69,70]. It also included novel items relevant to the COVID-19 pandemic, as described below.

#### Daily prosocial helping.

Prosocial helping items consisted of 11 items adapted from the Self-Report Altruism Scale [71]. Items measured engagement in specific helping behaviors, such as “picked up a fallen object for someone”, “held open a door”, “asked someone if they needed help”, or “helped with work/schoolwork”, and for whom the action was directed toward, including a family member, a friend, an acquaintance, or a stranger (multiple options could be selected). Following prior research, items were presented in a random order for each survey [67,68]. The total count of prosocial helping activities was summed for each day, and then an average daily helping score was calculated across the five days. For example, if a participant indicated they held open a door for a stranger and a friend every day they completed the survey, their average daily helping score would equal 2.

#### Daily social activity.

Daily social activity scores measured time spent engaged in both in-person and remote social activities. The seven in-person social activity items included face-to-face conversations with a spouse or partner, a family member, a close friend, others at a religious service, others at a group meeting, colleagues or co-workers, and strangers. The four remote social activity items included spending time talking on the phone, texting or email messaging, talking on video chat, and being on social media. These remote social items explicitly asked participants to indicate only their time spent for non-work purposes. Items were scored on a 6-point scale: 0 = “Didn’t do this”/ “Didn’t have this contact”, 1 = “Less than 30 minutes”, 2 = “30-59 minutes”, 3 = “1-1.5 hours”, 4 = “1.5-2 hours”, 5 = “Over 2 hours.”

#### Non-social activity items.

Non-social activity items were included to aid in examining the specificity of social and behavioral changes. Items included time spent in low-intensity physical activity (walking, stretching), moderate-high intensity physical activity (running, swimming, lifting), leisure (watching TV, reading for pleasure, browsing the internet), and cooking. Response options used the same 6-point scale as the social activity measures. Finally, a measure of time spent sleeping the previous night was included, which had response options in 1-hour intervals between anchor items of 0 = “Didn’t do this” and 12 = “12 or more hours”.

#### COVID-19 related factors.

We included four COVID-19-related self-report measures: self-reported social distancing behaviors, feelings of emotional isolation, feelings of physical isolation, and face mask usage. Social distancing behaviors were measured by responses to the question “How much did you socially distance today?”, which was reported using a visual analogue scale (VAS) ranging from 0 = “No social distancing” to 100 = “Maximal social distancing.” Feelings of emotional and physical isolation were measured by items “How much did you feel physically isolated today?” and “How much did you feel emotionally isolated today?”, which were reported using a VAS from 0 = “Not at all” to 100 = “Extremely.” Face mask usage was assessed by the question “How often did you wear a face mask when you were near other people who you do not live with today?”, to which participants responded on a 5-point scale: 1 = “Never,” 2 = “Rarely,” 3 = “Sometimes,” 4 = “Often,” or 5 = “Always.” The sixth response option, “I did not have contact with people I don’t live with today” was censored in analyses. We also measured three COVID-19-related variables using publicly available data: 1) county-level COVID-19 case rate, 2) county-level COVID-19 death rate, and 3) county-level COVID-19 vaccination rate. These measures all used a seven-day rolling county average based on participants’ ZIP codes and their specific dates of experience sampling.

#### Pain induction and measures.

During the baseline and follow-up sessions, acute pain was induced with the wall sit test (also known as the Roman chair test), an exercise that has been utilized for pain induction in multiple prior in-person studies [22,49–52], including studies testing the relationship between social outcomes and pain [22,50]. Participants held an unsupported seat-like position with their back against a wall for as long as possible. Advantages of the wall sit test include that it can be safely and effectively administered remotely and that outcomes using this test have previously been associated with various measures of social well-being, including social network size [22] and social bonding [50]. The wall sit test was chosen over commonly used laboratory pain induction methods (e.g., cold pressor, thermal stimulation, or pressure algometry) because those paradigms require specialized equipment and in-person administration, which were not feasible given the remote, video conference-based nature of the present study conducted during the COVID-19 pandemic. Ischemic exercise-based tasks such as the wall sit allow for standardized, self-terminated pain induction with only a timer and a wall, producing gradually increasing muscular discomfort that participants can safely tolerate or end at any point. This makes the task well-suited for online administration while preserving ecological validity and remaining consistent with prior research linking wall sit performance to social outcomes.

An image of the wall sit position was shown to participants, who then demonstrated their wall sit position to the researcher and performed a 5-second trial of the position. The researcher provided corrections on positioning when necessary. Participants were then asked to close their eyes once they began the wall sit, say “pain” when they first started to feel any pain at all, and then say “stop” when they could no longer bear the pain and/or when they fell to the ground. The number of seconds that the participant was able to hold the wall sit position indicated pain tolerance and the number of seconds that elapsed between start time and when the participant first said “pain” indicated pain threshold. Immediately after completing the wall sit test, participants received the online survey instruction, “Please rate the following items based on your experience during the wall sit test.” Subjective pain was measured on a scale with 10-point intervals ranging from 0 = “No pain” to 100 = “Worst pain imaginable.” Participants were then instructed, “Please describe the pain you just experienced during the wall sit test.” These qualitative descriptions were not analyzed and are not discussed here. All instructional scripts and the image of the wall sit position that was shown to participants are publicly available on Open Science Framework.

#### Statistical inference.

We applied an intent-to-treat approach such that all participants’ data, regardless of meditation training completion status, were included in analyses. Data analyses were carried out using STATA 15 and STATA 18. Group comparisons for continuous data were analyzed using two-tailed *t*-tests, and group comparisons for categorical data were analyzed using chi-squared analyses. We used Spearman’s rank correlation due to skewness in several social well-being and COVID-19-related variables. For our main hypotheses that planned to use two (meditation group: LKM/PMR) x two (time: baseline/follow-up) analyses of variance (ANOVA), linear mixed models were used instead because they could better handle covariates that differed between timepoints. Model results reported in the next section tested the effect of meditation group, time, and meditation group x time interaction on the dependent variable with random intercepts allowed for participants. We also ran supplemental models that included age, gender, level of education, and county-level COVID-19 case rates as covariates in linear mixed models for all main hypotheses, as well as average daily physical activity in analyses of acute pain tolerance and subjective pain. Age, level of education, and county-level COVID-19 case rates were mean-centered. Most results from these supplemental models with covariates were not significant, but all statistically significant coefficient relationships are described below.

Prior to conducting inferential tests, the distributional assumptions of each analytic strategy were evaluated. For independent-samples t-tests and ANOVA, normality of the outcome was observed within each group by inspecting histograms and Q–Q plots, and homogeneity of variance was assessed with Levene’s test. For linear mixed models, distributions of the residuals and best linear unbiased predictors for the random intercepts were approximately normal and we inspected residuals-versus-fitted plots for homoscedasticity and linearity. Potential outlier observations were checked using standardized residuals and Cook’s distance.

## Results

Participants in both groups completed similar numbers of meditation sessions on average (LKM: *M =* 87.6%, *SD* = 22.4%; PMR: *M* = 88.3%, *SD* = 16.6%; *t*(67) = 0.15, *p* = .881). The number of days between the baseline and follow-up session did not differ between groups (LKM: *M* = 36.88, *SD* = 2.69; PMR: *M* = 36.51, *SD* = 3.61; *t*(67) = 0.48, *p* = .634).

### Social support, loneliness, emotional isolation, and anxiety

**Fig 4:**
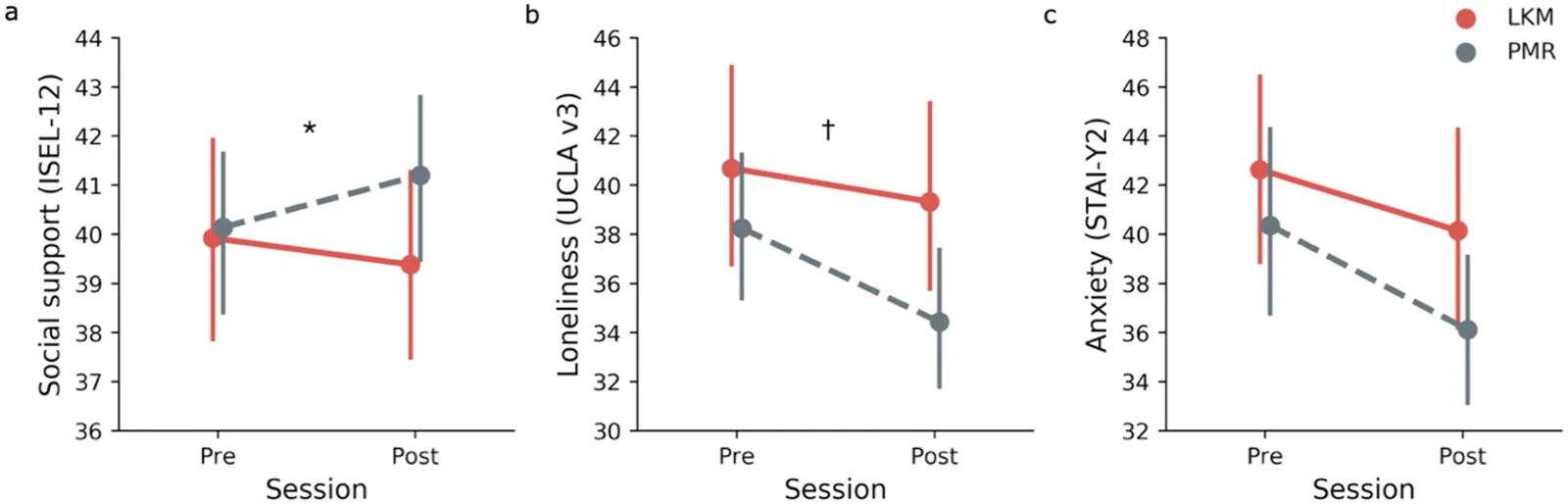
PMR was associated with greater social support relative to LKM. (a) Social support increased after PMR relative to LKM (*p* = .028). (b) Loneliness decreased between baseline and follow-up sessions (*p* = .003) and showed a trending group x time interaction with a greater reduction after PMR relative to LKM (*p* = .054). (c) Anxiety decreased between sessions (*p* = .002), but there was no significant group x time interaction (p = .209). *Note:* ^*^p < .05; ^†^*p* = 0.054.

Analyses to test the effect of the meditation training on social support found, contrary to hypotheses, that social support (ISEL-12 total score) increased after PMR relative to LKM (meditation group x time: *b* = −1.86, z = −2.20, *p* = .028; Fig 4a). There was not a significant main effect of meditation group (*b* = −0.22, *z* = −0.17, *p* = .868) or time (*b* = 0.85, *z* = 1.43, *p* = .153) on social support. Loneliness (UCLA v3) decreased between baseline and follow-up sessions (*b* = −3.26, *z* = −2.96, *p* = .003; Fig 4b), and there was a trending effect of meditation group x time interaction on loneliness (*b* = 3.02, *z* = 1.93, *p* = .054), indicating a greater reduction in loneliness after PMR relative to LKM. There was not a significant main effect of group on loneliness (*b* = 2.43, *z* = 0.96, *p* = .337). Neither meditation group nor time had a significant effect on emotional isolation (meditation group: *b* = 10.7, *z* = 1.43, *p* = .153; time: *b* = −1.60, *z* = −0.48, *p* = .633; meditation group x time: *b* = .081, *z* = 0.02, *p* = .986).

We conducted exploratory tests on changes in anxiety, which we expected to decrease after PMR. We had no hypothesis related to LKM. Results showed that anxiety (STAI-Y2) decreased across all participants between baseline and follow-up sessions (*b* = −3.73, *z* = −3.14, *p* = .002; Fig 4c), but there was no main effect of meditation group (b = 2.27, z = 0.88, p = .378) or interaction between meditation group and time (*b* = 2.13, *z* = 1.69, *p* = .209) on anxiety.

### Daily prosocial helping and social activity

Following the meditation training, there were no significant differences in daily prosocial helping (time: *b* = 0.00, *z* = 0.11, *p* = .915; meditation group: *b* = 0.07, *z* = 1.64, *p* = .102; meditation group x time: *b* = 0.01, *z* = 0.30, *p* = .767), daily in-person social activity (time: *b* = 0.02, *z* = 0.36, *p* = .719; meditation group: *b* = −0.08, *z* = −0.73, *p* = .462; meditation group x time: *b* = 0.04, *z* = 0.51, *p* = .608), or daily remote social activity (time: *b* = 0.02, *z* = 0.20, *p* = .842; meditation group: *b* = 0.26, *z* = 1.48, *p* = .139; meditation group x time: *b* = −0.11, *z* = −0.83, *p* = .407). Including county-level COVID-19 case rates and age as covariates impacted social activity such that above average county-level COVID-19 case rates were associated with decreased in-person social activity (*b* = −.005, *z* = −2.43, *p* = .015), while having an age above the mean age of the sample was associated with decreased remote social activity (*b* = −.025, *z* = −2.54, *p* = .011).

### Acute pain tolerance and subjective pain

**Fig 5:**
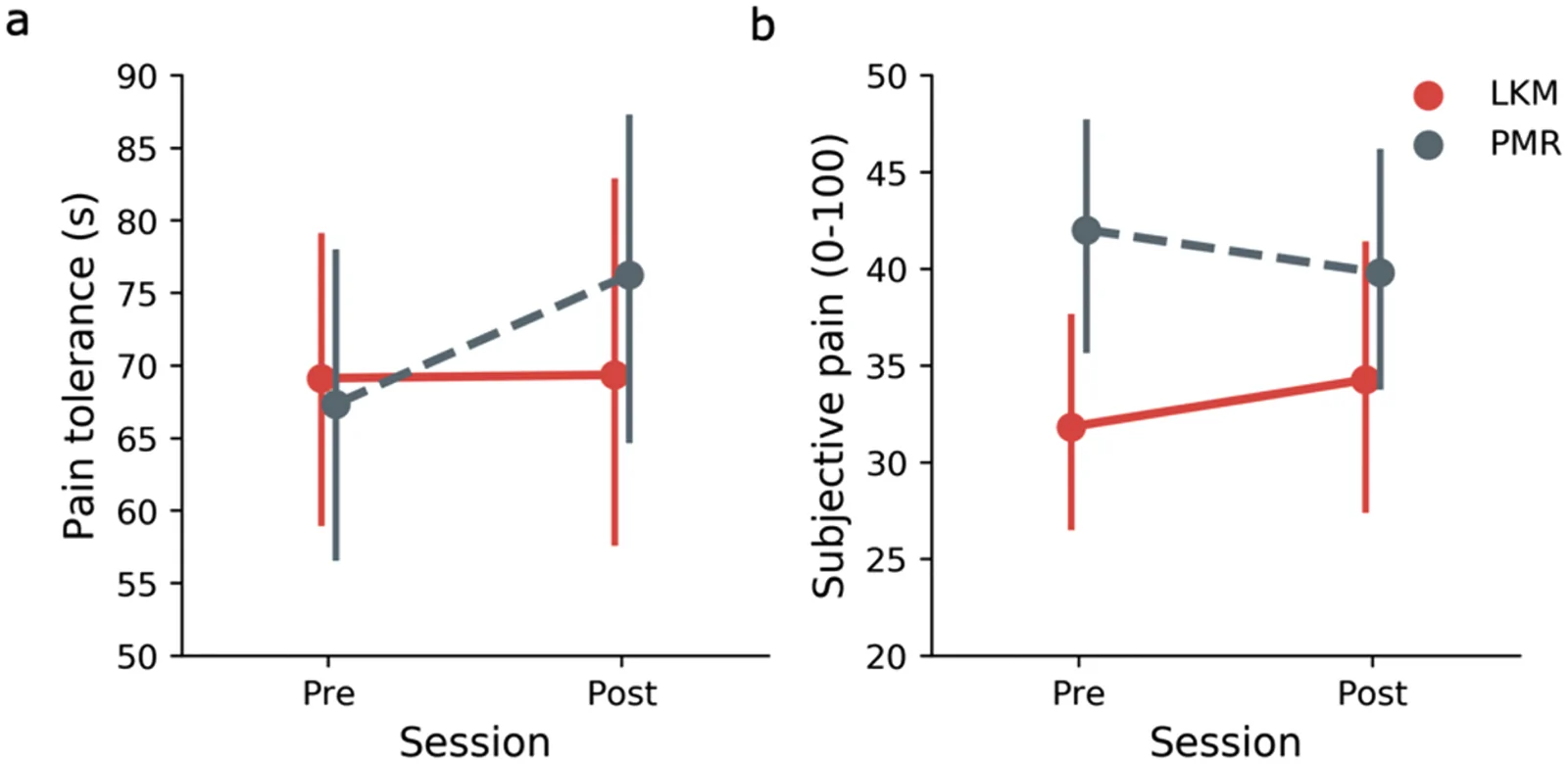
No significant effect of meditation on acute pain over time. Neither (a) pain tolerance nor (b) subjective pain were significantly affected over time by meditation.

Acute pain tolerance on the wall sit test increased by 8.5 s between baseline and follow-up sessions (*b* = 8.46, *z* = 2.09, *p* = .037; Fig 5a; Table 2), but there was no significant main effect of meditation group (*b* = 1.77, *z* = 0.22, *p* = .824) or a group x time interaction (*b* = −9.45, *z* = −1.63, *p* = .103). Average daily physical activity was marginally associated with pain tolerance at baseline (rho = .23, *p* = .051). However, including daily physical activity as a covariate did not meaningfully affect results. Subjective pain ratings after the wall sit were significantly higher for the PMR group (*b* = −10.19, *z* = −2.42, *p* = .015; Fig 5b), but there was no significant effect of time (*b* = −2.58, *z* = −0.91, *p* = .361) or a group x time interaction (*b* = 4.79, *z* = 1.18, *p* = .236) on subjective pain.

**Table 2:** Pre-to-Post Differences by Meditation Group (n = 69).

	Pre ^a^		Post ^a^		Cohen’s d [95% CI]	
*Social/ general well-being*	PMR	LKM	PMR	LKM	PMR	LKM
Social support (ISEL-12) ^b^	28.4 ± 4.82	28.5 ± 6.77	29.2 ± 5.09	27.38 ± 5.78	−.16 [−.63,.31]	.18 [−.30,.65]
Loneliness (UCLA v3) ^c^	37.57 ± 8.52	39.32 ± 12.84	34.43 ± 8.31	39.32 ± 11.89	.37 [−.10,.84]	0.0 [−.48,.48]
Anxiety (STAI-Y2) ^c^	39.71 ± 10.88	41.53 ± 11.70	36.11 ± 9.09	40.15 ± 11.45	.36 [−.11,.83]	.12 [−.36,.59]
COVID-19 emotional isolation ^d^	10 (0; 27.25)	19.5 (4; 49.2)	9 (.2; 27)	16.1 (1; 50.8)	.07 [−.40,.53]	.01 [−.47,.48]
COVID-19 physical isolation ^d^	7.6 (0; 34.25)	21.1 (2; 50.8)	11.6 (0; 31.6)	8.7 (0; 48)	−.12 [−.59,.35]	.26 [−.22,.73]
***Daily social activity***						
Daily social activity (in-person)	.91 ± .46	.83 ± .49	.93 ± .54	.89 ± .53	−.04 [−.50,.43]	−.11 [−.58,.37]
Daily social activity (non-in-person) ^d^	1.3 (.8, 1.8)	1.58 (1.2, 1.9)	1.3 (.85, 1.95)	1.4 (1.15, 2.25)	−.03 [−.50,.44]	.14 [−.34,.61]
Daily prosocial helping ^d^	.20 (.11,.31)	.29 (.15,.42)	.16 (.09,.33)	.29 (.16,.45)	−.01 [−.48,.45]	−.06 [−.53,.42]
***Pain***						
Pain tolerance ^c^	67.94 ± 33.74	69.88 ± 32.81	76.26 ± 35.86	69.36 ± 37.40	−.24 [−.71,.23]	.01 [−.46,.49]
Subjective pain ^e^	42.66 ± 17.61	31.65 ± 18.28	39.8 ± 18.09	34.27 ± 19.87	.16 [−.31,.63]	−.14 [−.62,.34]
Pain threshold ^f^	32.41 ± 21.69	34.19 ± 19.66	38.44 ± 23.96	33.06 ± 19.31	−.26 [−.74,.21]	.06 [−.43,.54]

^a^Values presented as mean ± standard deviation.

^b^Meditation group by time effect, *p* < .05

^c^Main effect of time, *p* < .05

^d^Due to skew, values presented as median (IQR)

^e^Main effect of meditation group, *p* < .05

^f^Two participants forgot to indicate time of pain onset during the baseline session, one participant did not complete the wall sit test at the follow-up session because of a knee injury, leaving *n* = 66.

**Fig 6:**
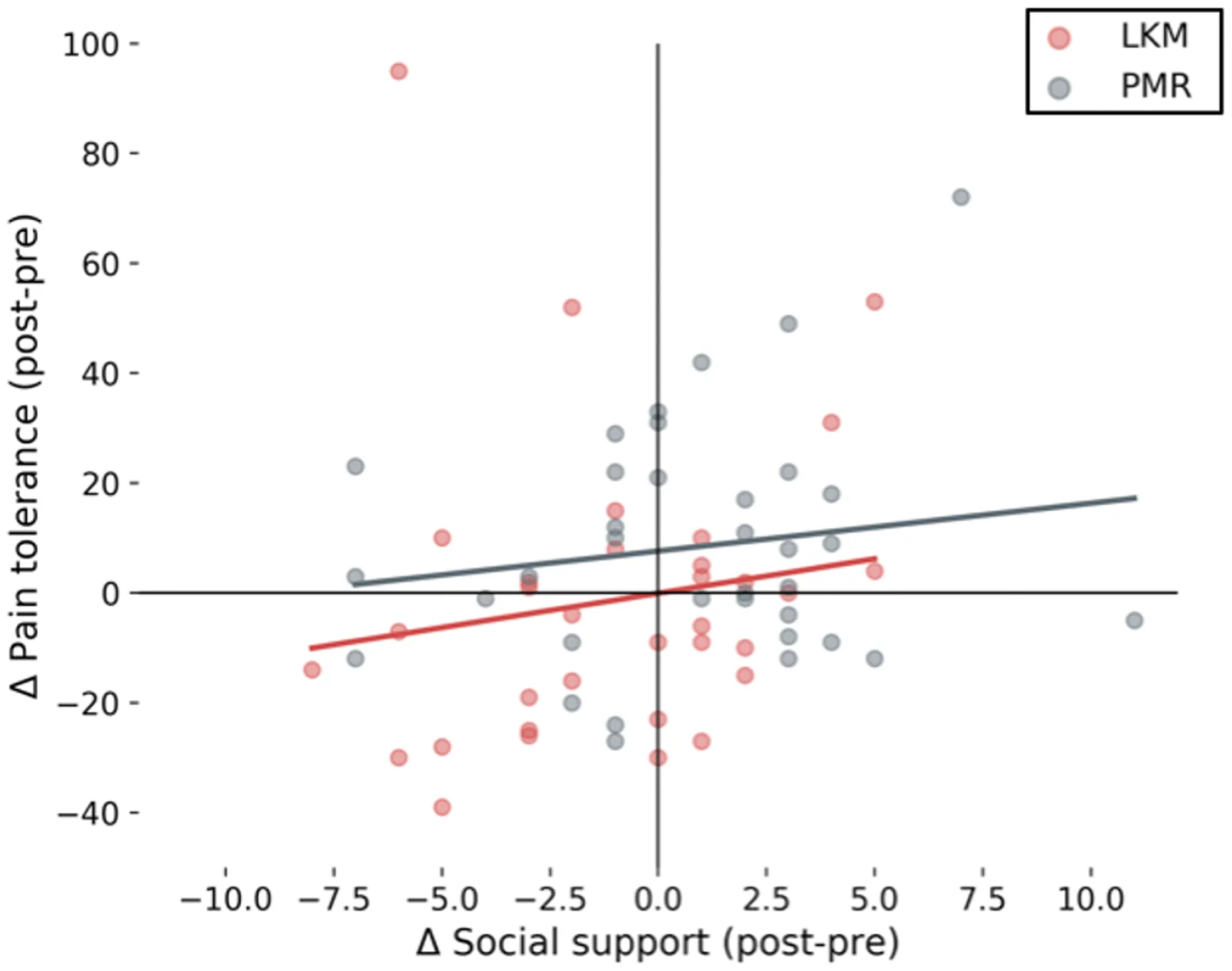
Correlation between change in social support with change in pain tolerance. Across groups, an increase in social support following meditation was associated with an increase in pain tolerance (rho = .249, *p* = .040).

Post hoc exploratory analyses evaluated change scores (follow-up – baseline) for acute pain tolerance and social support. Across participants, change in social support was positively associated with change in pain tolerance (rho = .249, *p* = .040, *n* = 69; Fig 6), suggesting that increased social support following (either) meditation may be associated with improved pain tolerance at follow-up. When evaluating this relationship within each group separately, correlations did not reach statistical significance (LKM: rho = .301, *p* = .089, *n* = 33; PMR: rho = .06, *p* = .727, *n* = 35).

### Baseline correlations among social support, pain, anxiety and COVID-19 related factors

There was a positive correlation between social support and pain tolerance at baseline (rho = .250, *p* = .032), consistent with prior literature using the wall sit test [22]. Neither baseline loneliness (rho = −.024, *p* = .837) nor anxiety (rho = −.006, *p* = .963) exhibited a significant relationship with baseline pain tolerance. Baseline pain tolerance showed no significant correlation with baseline subjective pain ratings (rho = .002, *p* = .986), in line with prior research comparing objectively measured pain tolerance and pain threshold to subjective reports of pain [72]. No significant correlations were observed between baseline pain tolerance, subjective pain, pain threshold and any of our COVID-19-related variables (see S1 Table). All analyses were preregistered unless otherwise specified as exploratory.

## Discussion

In this preregistered clinical trial, we examined the effects of an LKM intervention on social well-being and acute pain. Contrary to our hypotheses, we found that the active control meditation, PMR, increased perceptions of social support (*p* = .028) and had a trending effect on reducing loneliness (*p* = .054), while no change was observed following LKM. Neither meditation had a significant effect on acute pain tolerance or subjective pain over time. However, through exploratory analysis, we found that an increase in social support between baseline and follow-up timepoints was associated with increased pain tolerance across the sample, similar to what has been observed in prior work [22]. In sum, our findings suggest that interventions that improve social support could potentially benefit acute pain tolerance in healthy adults, but do not establish that brief, online LKM is effective for this purpose. Further research directly comparing LKM with other social-support-enhancing interventions could clarify relative efficacy.

It is possible that the contrast between prior literature on LKM and our findings, which did not yield the hypothesized positive effects of LKM on pain or social well-being, is due to methodological issues that are common in research on LKM [73–75]. Several systematic reviews have noted that studies on compassion-based meditation often use a poorly designed active control group or lack an active control group altogether, which leaves results vulnerable to positive expectancy effects [73–75]. Additionally, studies evaluating the prosocial effects of meditation have been criticized for an over-reliance on marginally significant results [73], dependence on small sample sizes [75], and high risk of experimenter bias, particularly when the teacher of the meditation in the study is listed as a co-author [73]. Although our design addresses some of methodological concerns raised about prior LKM research, it remains limited in other ways. For example, its reliance on a single experimental pain paradigm conducted only in healthy volunteers and the unique circumstances of the COVID-19 pandemic limit generalizability. These and other limitations discussed below suggest that the present null findings should be interpreted as one informative data point within an evolving literature rather than as definitive evidence.

Another possible reason we found no pain-reducing or positive social effects of LKM is that the benefits of a meditation program may be transient for new meditators. For example, one study involving a 21-day mindfulness meditation program found increases in positive emotions in the minutes to hours following practice but no evidence that this effect carried over to the following day [76]. In another study using an eight-week mindfulness-based meditation intervention, there were lasting improvements in pain acceptance and feelings of control over pain but not in reported pain itself [77]. We do not think that the reason neither meditation affected pain over time was because of the relatively short duration of meditation training, as studies show that two- to three-week meditation training interventions can effectively reduce subjective pain reports [78–80] and increase pain threshold [80]. For example, reported pain intensity decreased after participants completed a two-week training with only four 20-minute sessions of a Savoring Meditation, which like LKM focuses on generating positive emotions, but does so through instructing participants to recall a meaningful memory [78].

It is also important to consider that the unique social landscape during the early COVID-19 pandemic could have impacted the efficacy of LKM. The few studies that evaluated LKM during the beginning stages of the COVID-19 pandemic focused on specific populations that continued in-person work activities. For instance, among neonatal intensive care unit (NICU) nurses, a 4-week LKM intervention decreased compassion fatigue, a phenomenon in which caregivers experience reduced compassion and emotional exhaustion [81]. Among flight attendants, an 8-week LKM program increased subjective well-being, spirituality, and social presence (defined as the ability to represent oneself as a “real person” and connect with others through media communication) [82]. We did not find significant baseline correlations between social distancing and social support, loneliness, or anxiety. However, it is likely that our participants’ opportunities for in-person social connection were limited by factors related to the COVID-19 pandemic (e.g., a transition to a virtual work or academic environment, the prolonged closure of public community spaces), and it is possible that participants in the aforementioned studies benefited from LKM more than participants in our study because opportunities for in-person social connection were not as limited. Our LKM training may have increased our participants’ *desire* for social connection, but restrictions on opportunities for in-person social interaction could have led to a mismatch between preferred and actual degrees of social connection, which is central to perceptions of loneliness [83–85]. This potential explanation for why LKM did not improve social well-being among our participants is speculative, but it is worth considering due to the unprecedented nature of the social challenges posed by the COVID-19 pandemic.

We are not aware of any prior studies on PMR that are in line with our unexpected finding that PMR improved perceived social support. However, Heartfulness meditation, a form of meditation that is similar to PMR in that it focuses on sequentially relaxing different body parts, has been found to alleviate loneliness in high school students [86] and healthcare providers practicing during the COVID-19 pandemic [87]. Further research is necessary to determine if the effects of PMR and Heartfulness meditation are comparable, and if the beneficial effects of either meditation type could be explained by respite from pandemic-related stressors because positive distraction has improved emotional well-being in other research [88,89].

We chose to conduct the wall sit test remotely due to the COVID-19 pandemic, and to our knowledge, this is the first time that the wall sit test has been administered over video conference to experimentally induce pain. Baseline results confirmed that our online administration of the wall sit test effectively induced pain (see Table 2) and our indices of pain tolerance and threshold were strongly intercorrelated at baseline (rho = .71, *p* < 0.001). We experienced few problems or screening exclusions. Our study therefore provides evidence that pain can be rigorously measured remotely in the general population and builds upon a recent study demonstrating that a Cold Pressor Task, in which participants progressively immerse their hands into cold water, can successfully assess pain if performed at home [90]. Future work could evaluate the internet-based administration of other laboratory-based pain induction procedures, such as topical capsaicin delivery [91] or painful pressure induced by an inflatable blood pressure cuff [13,92]. In a recent comparison of human versus robot-guided quantitative sensory testing (QST), in-person social interactions between researchers and participants did not have significant effects on experimentally-induced pain, which further supports the idea that different experimental pain paradigms are robust enough to be used outside of a laboratory setting [93]. Importantly, the use of internet-based pain induction protocols could make pain research more feasible and more accessible to members of underrepresented populations [94].

While the remote administration of the wall sit test contributes to the novelty of our study, it also presents a few limitations. While numerous studies have employed the wall sit test as an in-person method of pain induction [22,49–52], future research is needed to verify the reliability of the wall sit test when conducted via video conference. While PMR served as a rigorous active control matched on format and expectancy, its own potential effects on reducing pain during the wall sit is a limitation since it may have obscured LKM’s impacts especially since the study did not include a passive control arm. In other words, the null between-group differences reported here could technically reflect two similarly effective interventions rather than the absence of an effect for either. Moreover, wall sit endurance has been found to be positively associated with self-rated physical fitness [22], and a related potential confound is that exercise itself can increase pain tolerance [95] and reduce pain sensitivity [96,97]. However, since participants reported no change in exercise across timepoints and we found no baseline association between reported physical activity and pain tolerance, we remain confident that exercise-induced effects did not influence our results.

We also must consider several limitations that are unrelated to the LKM intervention or wall sit test. Prior research shows that survey respondents tend to overreport behaviors that are likely to be viewed favorably by others [98,99], including behaviors that we assessed, such as physical activity [98]. Some studies suggest that social distancing [100] and face mask usage [101] are also overreported, yet other recent research indicates that self-reports of COVID-19-related health behaviors accurately reflect objective measures [102,103]. In regard to our assessment of social distancing, specifically, it is also important to note that because we did not specifically define “social distancing,” our results may have been affected by differences in participants’ subjective interpretations of the term. Overall, our results should be considered context-specific, as it is difficult to fully distinguish between changes due to improved external factors (e.g., improvements in COVID-19 case and vaccination rates over time) versus the meditation training. Finally, while our sample includes adults throughout the continental United States, it was skewed towards young participants from urban areas who have internet access and overrepresents females.

Despite these limitations, the present research advances our understanding of the interplay between meditation, social well-being, and pain. Our findings extend the current literature on LKM to an online sample of the general population, studied during the unique circumstance of the early COVID-19 pandemic.

## Supporting information

S1 Table Pairwise correlations at baseline. (PDF)

S1 CONSORT Checklist CONSORT 2025 checklist of information to include when reporting a randomized trial. (PDF)
