# Supplementary material for: Assessing the effect of Loving-Kindness Meditation on pain and social well-being using a randomized controlled trial design
Source: PLoS One. 2026 Sep 16;21(9):e0357888. doi: 10.1371/journal.pone.0357888 (PMC13580951; doi:10.1371/journal.pone.0357888)
Supplement: Supplementary file 1 [file pone.0357888.s001.pdf]

Supporting Information

S1 Table. Pairwise correlations at baseline (n = 74[a])

	1	2	3	4	5	6	7	8	9	10	11	12	13	14	15	16	17	18	19	20
**1. Age**	-																			
**2. Sex**	-.09	-																		
**3. Education**	.52***	-.06	-																	
**4. Pain Tolerance**	.00	.04	-.03	-																
**5. Subjective Pain**	-.07	-.13	.08	.00	-															
**6. Pain Threshold**	.02	-.05	.02	.71***	-.11	-														
**7. Social Support**	.21	.00	.16	.25*	.10	.20	-													
**8. Loneliness**	-.13	.09	-.20	-.02	-.24*	.03	-.76***	-												
**9. Anxiety**	-.20	.26*	-.16	-.01	-.16	.07	-.49***	.69***	-											
**10. Prosocial Helping**	.02	.00	.00	-.23	.06	-.17	.26*	-.18	-.03	-										
**11. In-Person Social Activity**	.02	-.01	.18	.07	.04	-.02	.28*	-.17	-.05	.55***	-									
**12. Remote Social Activity**	-.28*	.15	.02	-.16	.02	-.09	-.17	.09	.24*	.10	.23	-								
**13. Physical Activity**	.17	-.07	.14	.23	-.13	.12	.28*	-.22	-.26*	.29*	.39***	-.06	-							
**14. COVID-19 Cases**	-.09	.16	-.22	.07	-.05	.12	-.11	.11	.06	-.27*	-.35***	-.06	-.40***	-						
**15. Social Distancing**	.06	.01	-.01	-.10	.03	-.06	-.11	-.05	-.03	-.17	-.29*	-.10	-.05	.28*	-					
**16. COVID-19 Deaths**	-.03	.32**	.00	-.11	-.12	-.02	-.09	.13	.17	-.11	-.16	-.02	-.38***	.60***	.15	-				
**17. Face Mask Usage**	.00	.08	-.02	-.07	.03	-.04	-.08	-.04	-.01	-.21	-.47***	.06	-.28*	.37***	.57***	.15	-			
**18. COVID-19 Vaccination**	.16	-.36***	.07	-.19	.07	-.19	.05	-.13	-.15	.21	.24*	-.04	.35***	-.77***	-.18	-.65***	-.39***	-		
**19. Emotional Isolation**	-.29*	.20	-.19	-.12	-.06	-.08	-.57***	.62***	.65***	-.19	-.24*	.17	-.22	.11	.19	.15	.16	-.29*	-	
**20. Physical Isolation**	-.27*	.26*	-.15	-.08	-.06	-.06	-.50***	.51***	.57***	-.28*	-.27*	.25*	-.24*	.24*	.35***	.27*	.33***	-.37***	.88***	

Note: *p <.05, **p <.01, ***p <.005

[a]Two participants forgot to indicate the time of pain onset, leaving n = 72 for pain threshold (item 6). Two participants did not complete any of the daily experience sampling items and one participant completed all daily experience sampling items except for face mask usage, leaving n = 72 for items 10-16 and 18-20, and n = 71 for item 17.
