# Supplementary material for: Assessing the effect of Loving-Kindness Meditation on pain and social well-being using a randomized controlled trial design
Source: PLoS One. 2026 Sep 16;21(9):e0357888. doi: 10.1371/journal.pone.0357888 (PMC13580951; doi:10.1371/journal.pone.0357888)
Supplement: Supplementary file 3 [file pone.0357888.s003.docx]

**Manuscript Number PONE-D-25-57691
"Assessing the effect of Loving-Kindness Meditation on pain and social well-being using a randomized controlled trial design"**

1. Brief summary

This study is a randomized controlled trial comparing a 4-week online Loving-Kindness Meditation (LKM) program with Progressive Muscle Relaxation (PMR) in healthy adults, evaluating social well-being and experimentally induced acute pain using a wall sit task. Contrary to the hypothesis that LKM would enhance social well-being and thereby improve pain tolerance and reduce subjective pain, no significant between-group differences were observed on pain-related outcomes, and increases in perceived social support and a trend toward reduced loneliness were observed only in the PMR group. In addition, the trial was conducted entirely online during the early phase of the COVID 19 pandemic, which constitutes an important contextual feature of this study.

2. Strengths

- The authors increased the transparency and reproducibility of the study by registering the trial on ClinicalTrials.gov and preregistering the hypotheses, design, and analysis plan on OSF.
- The use of stratified block randomization by sex and age with envelope-based allocation concealment, together with an ITT approach and linear mixed models, represents a relatively rigorous design and analysis for an RCT.
- The LKM and PMR intervention protocols (4 weeks, 6 sessions per week, 15 minutes per session) are well standardized, and adherence is carefully monitored via Qualtrics by tracking session duration and completion status.
- Social well-being and its context are assessed in a multidimensional way (perceived social support, loneliness, anxiety, daily social and non-social activities, and COVID 19–related indicators), providing rich data to interpret null and partially positive findings.
- Finally, the introduction of a wall sit task delivered via videoconference as a remote pain-induction paradigm is a methodological strength, demonstrating that acute pain can be measured in a reasonably reliable way in an online setting.

3. Major comments

1) Abstract

It would be helpful if the abstract explicitly stated that this study uses a randomized controlled trial (RCT) design. If possible, in line with the CONSORT for Abstracts recommendations, I would also suggest adding one brief sentence describing the level of blinding (e.g., whether the trial was open-label) and the basic randomization procedure.

In addition, since the abstract mentions the COVID‑19 context, it may help readers interpret the findings if you could add one sentence indicating how this period might plausibly have influenced social support or pain reporting (e.g., by restricting in-person social contact or increasing background stress)

2) Introduction

The Introduction clearly presents the study hypothesis that LKM affects pain via improvements in social well-being. However, adding a conceptual figure at the end of the Introduction that schematically depicts this hypothesized pathway would, in my view, help readers grasp the overall structure of the study and its key variables at a glance.

In addition, while LKM is introduced in the Introduction in relatively considerable detail as an intervention with various positive effects on social, emotional, and pain-related outcomes, PMR is mentioned only briefly, mainly to note that it has been shown to reduce anxiety but has not been reported to enhance social connectedness. It would therefore be helpful to provide a clearer theoretical rationale for why PMR was selected as the active control intervention in this study.

In particular, for readers who may not be familiar with either LKM or PMR, the fact that the pain induction method used in this study is the wall sit test—which imposes a load on the lower body muscles—may intuitively lead them to assume that PMR, which directly targets muscular relaxation, could be more effective in reducing pain. Considering this point, it would be helpful if the Introduction more clearly explained the theoretical reasoning for why PMR was considered an appropriate control condition in this context.

As this is my personal interpretation, clarifying the authors’ intended rationale for this comparison would likely help readers better understand the logic underlying the study.

3) Methods

I consider it a strength that the meditation sessions were delivered via pre-recorded audio through Qualtrics and that session duration and completion were tracked in real time, as this allows for an objective assessment of intervention adherence. However, this approach can only confirm that the audio file was played to the end, and it does not guarantee that participants actually followed the meditation instructions and remained focused on the practice during that time. If possible, in future studies, it may be useful to include brief self-report measures of practice engagement (e.g., perceived immersion, distraction) so that not only the quantity but also the quality of meditation practice can be evaluated.

In the Methods section, the authors describe using the wall‑sit test as a remotely administered experimental pain task, but the theoretical and practical reasons for choosing the wall‑sit over other possible pain induction methods are not fully elaborated. Providing a more detailed rationale for this choice would, in my view, help readers better understand and evaluate the suitability of the selected pain paradigm.

4) Discussion

The final sentence of the first paragraph of the Discussion (“there may be more effective methods than meditation”) carries a risk of being interpreted as a general conclusion that meditation as a whole might be less effective than other interventions, beyond the specific context of this trial (healthy adults, a 4‑week online intervention, and the early phase of the COVID‑19 pandemic). Given that the results of the present study (1) showed no clear effects of either LKM or PMR on pain outcomes, (2) found only partial evidence of increased social support and a trend toward reduced loneliness in the PMR group, and (3) are based on a relatively modest sample size (n = 69), it would seem more appropriate to temper this sentence so that the conclusion is clearly restricted to the particular conditions and protocols examined in this study.

I find it very helpful that the Discussion highlights several methodological limitations of prior LKM studies, such as small sample sizes, weakly designed control conditions, over-reliance on marginally significant findings, and potential experimenter bias when the meditation teacher is a co‑author. At the same time, the present study also has its own constraints, including a healthy volunteer sample, the use of a single experimental pain paradigm, and the unique circumstances of the COVID‑19 pandemic. More explicitly acknowledging these limitations of the current study alongside the critique of the existing LKM literature could help reduce the risk that readers interpret the message as “prior studies were methodologically weak, and only our more rigorous design failed to find effects.”

4. Minor comments

In the Methods section (page 6, line 119), the phrase “contradictions to safe participation” appears to be a typographical error and likely should read “contraindications to safe participation.” I would also recommend a careful proofread of the manuscript to check for and correct any remaining minor wording or spelling issues prior to publication.
