# Supplementary material for: Assessing the effect of Loving-Kindness Meditation on pain and social well-being using a randomized controlled trial design
Source: PLoS One. 2026 Sep 16;21(9):e0357888. doi: 10.1371/journal.pone.0357888 (PMC13580951; doi:10.1371/journal.pone.0357888)
Supplement: Supplementary file 4 [file pone.0357888.s005.docx]

Katherine O’Connell

May 20, 2026

Assessing the effect of Loving-Kindness Meditation on pain and social well-being using a randomized controlled trial design

**Response to Reviewer Comments**

We thank the Editor and the two reviewers for their thorough and constructive feedback on our manuscript. The comments have improved the clarity and transparency of the manuscript. Below we respond to each comment in turn. Reviewer comments are in italics and our responses follow. All revisions have been implemented using tracked changes in the revised manuscript; section, paragraph, and figure references below refer to the revised version.

# Reviewer 1

***Comment 1.1.*** *Abstract: The authors stated that “We predicted that, relative to the PMR active control...” Predicted must be changed to hypothesize.*

**Response:** We have changed “predicted” to “hypothesized” in the Abstract and Discussion.

***Comment 1.2.*** *Sample size: There must be a clearly defined section titled sample size. What informed the treatment effect size of partial eta-squared of 0.02? A reference or justification must be provided. What was the power of the study and the margin of error used in estimating the sample size? Did you adjust the sample size for non-response rate?*

**Response:** We expanded the Sample size subsection of the Methods to present the a priori power analysis that we pre-registered on the Open Science Framework. Specifically, we now report that the primary power calculation targeted a treatment effect of partial η² = 0.02 on change in pain tolerance in a 2 (meditation group) × 2 (time) repeated-measures design, assuming a correlation between repeated measures of r = .75. This target effect size was selected as a conservative estimate consistent with the modest effects reported in prior research linking social well-being interventions to experimental pain outcomes in healthy adults (e.g., Roberts et al., 2015; Johnson & Dunbar, 2016)

An a priori analysis in G*Power yielded a required total sample of N = 68 (n = 34 per arm) to achieve 90% power at a two-tailed α = .05. With N = 68, we also expected greater than 80% power to detect group differences on self-reported social well-being outcomes given prior effect sizes of Cohen’s d = 0.83–1.45, using a Bonferroni-adjusted α = .05/4 = .0125 to accommodate four planned comparisons. To accommodate an anticipated attrition and non-response rate of approximately 10%, target enrollment was set at 74.

This target effect size was selected as a conservative estimate consistent with the modest effects reported in prior research linking social well-being interventions to experimental pain outcomes in healthy adults (e.g., Roberts et al., 2015; Johnson & Dunbar, 2016)

We did not specify a separate margin of error, but a study powered to detect a minimum effect of partial η² = 0.02 will tend to produce confidence intervals that are reasonably narrow. The achieved N = 69 (75 enrolled, 6 lost to attrition) met the pre-registered target.

***Comment 1.3.*** *Under study design, provide the name of the specific study design that was used before you continue with the detailed description of the study.*

**Response:** We now explicitly refer to the design as a “two-arm, parallel-group randomized controlled trial” consistently in the Methods.

***Comment 1.4.*** *There must be a section titled outcome measures that describes each key outcome measure… and how it was measured… There should be another section titled“Description of the intervention.”*

**Response:** We added two new subsection headings in the Methods: “Description of the Intervention” (introducing the LKM and PMR content) and “Outcome Measures” (introducing the subjective social well-being measures and pain outcomes). We agree the edits clarify the organization.

***Comment 1.5.*** *Wondering why two different versions of the same statistical software were used in analyzing the data.*

**Response:** The initial analyses (conducted at the time of pre-registration and primary analysis) were run in STATA 15, which was the version available to the analytic team at that time. Follow-up analyses and output verification during manuscript revision were conducted in STATA 18 after a scheduled upgrade.

***Comment 1.6.*** *What key assumptions were considered for the use of the two-tailed t-test, analyses of variance (ANOVA), and linear mixed models, and how were these assumptions tested to warrant their use? These details must be provided in the manuscript*

**Response:** We added a new paragraph at the end of the Statistical inference subsection describing the assumption checks. In brief: normality within each group was assessed by inspecting histograms, Q–Q-plots and homogeneity of variance was assessed with Levene’s test. For linear mixed models, we examined distributions of residuals for normality, and residuals-versus-fitted plots for homoscedasticity and linearity. Potential outlier observations were checked using standardized residuals and Cook’s distance.

# Reviewer 2

***Comment 2.1.*** *It would be helpful if the abstract explicitly stated that this study uses a randomized controlled trial (RCT) design. If possible, in line with the CONSORT for Abstracts recommendations, I would also suggest adding one brief sentence describing the level of blinding (e.g., whether the trial was open-label) and the basic randomization procedure.*

*In addition, since the abstract mentions the COVID 19 context, it may help readers interpret the findings if you could add one sentence indicating how this period might plausibly have influenced social support or pain reporting (e.g., by restricting in-person social contact or increasing background stress)*

**Response:** We have revised the Abstract to (a) state explicitly that this is a “two-arm, parallel-group randomized controlled trial design with stratified block randomization and a double-blind baseline session”, and (b) note that data were collected during the early phase of the COVID-19 pandemic, when social well-being and daily activity were atypical.

***Comment 2.2.*** *…adding a conceptual figure at the end of the Introduction that schematically depicts this hypothesized pathway would, in my view, help readers grasp the overall structure of the study…* *It would therefore be helpful to provide a clearer theoretical rationale for why PMR was selected as the active control intervention in this study…*

**Response:** We added a new conceptual figure (Figure 1) and an accompanying caption summarizing the hypothesized pathway by which LKM is expected to influence acute pain tolerance, pain threshold, and subjective pain through improvements in social well-being (social support, loneliness, prosocial activity and social activity), with PMR serving as an active control matched on format, dose, attention, and relaxation but not hypothesized to operate through a social pathway.

We also added a short paragraph to the Introduction describing the rationale for selecting PMR. PMR is a widely used relaxation protocol that shares with LKM the features of guided audio, structured practice, comparable time commitment, focused attention, and a general relaxation response, but does not have a social or affective focus. These features make PMR a strong active control for isolating the specifically social-affective components of LKM.

Related to these changes, we added a sentence about limitations to the Discussion, “While PMR served as a rigorous active control matched on format and expectancy, its own potential effects on reducing pain during the wall sit is a limitation since it may have obscured LKM’s impacts especially since the study did not include a passive control arm. In other words, the null between-group differences reported here could technically reflect two similarly effective interventions rather than the absence of an effect for either.”

***Comment 2.3.*** *…this approach can only confirm that the audio file was played to the end, and it does not guarantee that participants actually followed the meditation instructions and remained focused… the theoretical and practical reasons for choosing the wall sit over other possible pain induction methods are not fully elaborated.*

**Response:** We now explain the rationale for choosing the wall sit test over alternative laboratory pain paradigms (cold pressor, thermal stimulation, pressure algometry), which require specialized equipment and in-person administration and were therefore not feasible given the remote, videoconference-based design and the COVID-19 context. We also note that ischemic, exercise-based tasks such as the wall sit allow for standardized, self-terminated pain induction with minimal equipment and preserve ecological validity, and that pain outcomes in the wall sit have previously been linked to social outcomes. In the newly added Limitations paragraph in the Discussion, we now also acknowledge that participant engagement with session audio was indexed primarily through self-report and that future work should incorporate more objective engagement metric tests.

***Comment 2.4.*** *The final sentence of the first paragraph of the Discussion (“there may be more effective methods than meditation”) carries a risk of being interpreted as a general conclusion… it would seem more appropriate to temper this sentence so that the conclusion is clearly restricted to the particular conditions and protocols examined in this study.*

**Response:** We agree and have revised the Discussion accordingly. The revised statement now reads, “In sum, our findings suggest that interventions that improve social support could potentially benefit acute pain tolerance in healthy adults, but do not establish that brief, online LKM is effective for this purpose. Further research directly comparing LKM with other social-support-enhancing interventions could clarify relative efficacy.”

***Comment 2.4. (continued) …****the present study also has its own constraints, including a healthy volunteer sample, the use of a single experimental pain paradigm, and the unique circumstances of the COVID 19 pandemic. More explicitly acknowledging these limitations of the current study alongside the critique of the existing LKM literature could help reduce the risk that readers interpret the message as “prior studies were methodologically weak, and only our more rigorous design failed to find effects.”*

**Response:** We added the following text to the Discussion, “Although our design addresses some of methodological concerns raised about prior LKM research, it remains limited in other ways. For example, its reliance on a single experimental pain paradigm conducted only in healthy volunteers and the unique circumstances of the COVID-19 pandemic limit generalizability. These and other limitations discussed below suggest that the present null findings should be interpreted as one informative data point within an evolving literature rather than as definitive evidence.”

***Comment 2.5.*** *Minor: “contradictions” should read “contraindications”.*

**Response:** Corrected.
