# Supplementary material for: Assessing the effect of Loving-Kindness Meditation on pain and social well-being using a randomized controlled trial design
Source: PLoS One. 2026 Sep 16;21(9):e0357888. doi: 10.1371/journal.pone.0357888 (PMC13580951; doi:10.1371/journal.pone.0357888)
Supplement: Peer review history

# Peer review: Decision Letter 0

**DOI:** 10.1371/journal.pone.0357888.r001

**Authors:** Won Ju Hwang

23 Mar 2026

Dear Dr. O'Connell,

Thank you for submitting your manuscript to PLOS ONE. After careful consideration, we feel that it has merit but does not fully meet PLOS ONE’s publication criteria as it currently stands. Therefore, we invite you to submit a revised version of the manuscript that addresses the points raised during the review process.

Please submit your revised manuscript by May 07 2026 11:59PM. If you will need more time than this to complete your revisions, please reply to this message or contact the journal office at plosone@plos.org. When you're ready to submit your revision, log on to https://www.editorialmanager.com/pone/ and select the 'Submissions Needing Revision' folder to locate your manuscript file.

- A letter that responds to each point raised by the academic editor and reviewer(s). You should upload this letter as a separate file labeled 'Response to Reviewers'.
- A marked-up copy of your manuscript that highlights changes made to the original version. You should upload this as a separate file labeled 'Revised Manuscript with Track Changes'.
- An unmarked version of your revised paper without tracked changes. You should upload this as a separate file labeled 'Manuscript'.

If you would like to make changes to your financial disclosure, please include your updated statement in your cover letter. Guidelines for resubmitting your figure files are available below the reviewer comments at the end of this letter.

If applicable, we recommend that you deposit your laboratory protocols in protocols.io to enhance the reproducibility of your results. Protocols.io assigns your protocol its own identifier (DOI) so that it can be cited independently in the future. For instructions see: https://journals.plos.org/plosone/s/submission-guidelines#loc-laboratory-protocols. Additionally, PLOS ONE offers an option for publishing peer-reviewed Lab Protocol articles, which describe protocols hosted on protocols.io. Read more information on sharing protocols at https://plos.org/protocols?utm_medium=editorial-email&utm_source=authorletters&utm_campaign=protocols.

We look forward to receiving your revised manuscript.

Kind regards,

Won Ju Hwang

Academic Editor

PLOS One

Journal requirements:

When submitting your revision, we need you to address these additional requirements.

1. Please ensure that your manuscript meets PLOS ONE's style requirements, including those for file naming. The PLOS ONE style templates can be found at

https://journals.plos.org/plosone/s/file?id=wjVg/PLOSOne_formatting_sample_main_body.pdf and

https://journals.plos.org/plosone/s/file?id=ba62/PLOSOne_formatting_sample_title_authors_affiliations.pdf

2. Thank you for stating the following financial disclosure:

“Research reported in this publication was supported by the National Center for Complementary and Integrative Health (NCCIH) and the National Center For Advancing Translational Sciences (NCATS) of the National Institutes of Health under award numbers 5F31AT010423 and TL1TR001431 to KO. Resources were also supported by the National Science Foundation through grant number 1729406 to AAM. The content is solely the responsibility of the authors and does not necessarily represent the official views of the National Institutes of Health or National Science Foundation.”

Please state what role the funders took in the study.  If the funders had no role, please state: "The funders had no role in study design, data collection and analysis, decision to publish, or preparation of the manuscript."

If this statement is not correct you must amend it as needed.

Please include this amended Role of Funder statement in your cover letter; we will change the online submission form on your behalf.

3. Please include your full ethics statement in the ‘Methods’ section of your manuscript file. In your statement, please include the full name of the IRB or ethics committee who approved or waived your study, as well as whether or not you obtained informed written or verbal consent. If consent was waived for your study, please include this information in your statement as well.

4. Please include captions for your Supporting Information files at the end of your manuscript, and update any in-text citations to match accordingly. Please see our Supporting Information guidelines for more information: http://journals.plos.org/plosone/s/supporting-information.

If the reviewer comments include a recommendation to cite specific previously published works, please review and evaluate these publications to determine whether they are relevant and should be cited. There is no requirement to cite these works unless the editor has indicated otherwise.

Additional Editor Comments:

PONE-D-25-57691

"Assessing the effect of Loving-Kindness Meditation on pain and social well-being using a randomized controlled trial design"

Original Submission

Would you please revised the manuscript according to the reviewers?

(Reviewer 1)

Here are my comments

1. Abstract: The authors stated that “We predicted that, relative to the PMR active control...” Predicted must be changed to hypothesize.

2. Sample size: There must be a clearly defined section titled sample size. What informed the treatment effect size of partial eta-squared of 0.02? A reference or justification must be provided. What was the power of the study and the margin of error used in estimating the sample size? Did you adjust the sample size for non-response rate?

3. Under study design, provide the name of the specific study design that was used before you continue with the detailed description of the study. Eg, this study employed an RCT design....

4. There must be a section titled outcome measures that describes each key outcome measure (primary and secondary outcomes if applicable) and how it was measured. The information is all lumped together, making it difficult to follow the manuscript. There should be another section titled “Description of the intervention”.

5. Wondering why two different versions of the same statistical software were used in analysing the data. Any explanation

6. What key assumptions were considered for the use of the two-tailed t-test, analyses of variance (ANOVA), and linear mixed models, and how were these assumptions tested to warrant their use? These details must be provided in the manuscript

Reviewer 2

1. Brief summary

This study is a randomized controlled trial comparing a 4-week online Loving-Kindness Meditation (LKM) program with Progressive Muscle Relaxation (PMR) in healthy adults, evaluating social well-being and experimentally induced acute pain using a wall sit task. Contrary to the hypothesis that LKM would enhance social well-being and thereby improve pain tolerance and reduce subjective pain, no significant between-group differences were observed on pain-related outcomes, and increases in perceived social support and a trend toward reduced loneliness were observed only in the PMR group. In addition, the trial was conducted entirely online during the early phase of the COVID 19 pandemic, which constitutes an important contextual feature of this study.

2. Strengths

� The authors increased the transparency and reproducibility of the study by registering the trial on ClinicalTrials.gov and preregistering the hypotheses, design, and analysis plan on OSF.

� The use of stratified block randomization by sex and age with envelope-based allocation concealment, together with an ITT approach and linear mixed models, represents a relatively rigorous design and analysis for an RCT.

� The LKM and PMR intervention protocols (4 weeks, 6 sessions per week, 15 minutes per session) are well standardized, and adherence is carefully monitored via Qualtrics by tracking session duration and completion status.

� Social well-being and its context are assessed in a multidimensional way (perceived social support, loneliness, anxiety, daily social and non-social activities, and COVID 19–related indicators), providing rich data to interpret null and partially positive findings.

� Finally, the introduction of a wall sit task delivered via videoconference as a remote pain-induction paradigm is a methodological strength, demonstrating that acute pain can be measured in a reasonably reliable way in an online setting.

3. Major comments

1) Abstract

It would be helpful if the abstract explicitly stated that this study uses a randomized controlled trial (RCT) design. If possible, in line with the CONSORT for Abstracts recommendations, I would also suggest adding one brief sentence describing the level of blinding (e.g., whether the trial was open-label) and the basic randomization procedure.

In addition, since the abstract mentions the COVID 19 context, it may help readers interpret the findings if you could add one sentence indicating how this period might plausibly have influenced social support or pain reporting (e.g., by restricting in-person social contact or increasing background stress)

2) Introduction

The Introduction clearly presents the study hypothesis that LKM affects pain via improvements in social well-being. However, adding a conceptual figure at the end of the Introduction that schematically depicts this hypothesized pathway would, in my view, help readers grasp the overall structure of the study and its key variables at a glance.

In addition, while LKM is introduced in the Introduction in relatively considerable detail as an intervention with various positive effects on social, emotional, and pain-related outcomes, PMR is mentioned only briefly, mainly to note that it has been shown to reduce anxiety but has not been reported to enhance social connectedness. It would therefore be helpful to provide a clearer theoretical rationale for why PMR was selected as the active control intervention in this study.

In particular, for readers who may not be familiar with either LKM or PMR, the fact that the pain induction method used in this study is the wall sit test—which imposes a load on the lower body muscles—may intuitively lead them to assume that PMR, which directly targets muscular relaxation, could be more effective in reducing pain. Considering this point, it would be helpful if the Introduction more clearly explained the theoretical reasoning for why PMR was considered an appropriate control condition in this context.

As this is my personal interpretation, clarifying the authors’ intended rationale for this comparison would likely help readers better understand the logic underlying the study.

3) Methods

I consider it a strength that the meditation sessions were delivered via pre-recorded audio through Qualtrics and that session duration and completion were tracked in real time, as this allows for an objective assessment of intervention adherence. However, this approach can only confirm that the audio file was played to the end, and it does not guarantee that participants actually followed the meditation instructions and remained focused on the practice during that time. If possible, in future studies, it may be useful to include brief self-report measures of practice engagement (e.g., perceived immersion, distraction) so that not only the quantity but also the quality of meditation practice can be evaluated.

In the Methods section, the authors describe using the wall sit test as a remotely administered experimental pain task, but the theoretical and practical reasons for choosing the wall sit over other possible pain induction methods are not fully elaborated. Providing a more detailed rationale for this choice would, in my view, help readers better understand and evaluate the suitability of the selected pain paradigm.

4) Discussion

The final sentence of the first paragraph of the Discussion (“there may be more effective methods than meditation”) carries a risk of being interpreted as a general conclusion that meditation as a whole might be less effective than other interventions, beyond the specific context of this trial (healthy adults, a 4 week online intervention, and the early phase of the COVID 19 pandemic). Given that the results of the present study (1) showed no clear effects of either LKM or PMR on pain outcomes, (2) found only partial evidence of increased social support and a trend toward reduced loneliness in the PMR group, and (3) are based on a relatively modest sample size (n = 69), it would seem more appropriate to temper this sentence so that the conclusion is clearly restricted to the particular conditions and protocols examined in this study.

I find it very helpful that the Discussion highlights several methodological limitations of prior LKM studies, such as small sample sizes, weakly designed control conditions, over-reliance on marginally significant findings, and potential experimenter bias when the meditation teacher is a co author. At the same time, the present study also has its own constraints, including a healthy volunteer sample, the use of a single experimental pain paradigm, and the unique circumstances of the COVID 19 pandemic. More explicitly acknowledging these limitations of the current study alongside the critique of the existing LKM literature could help reduce the risk that readers interpret the message as “prior studies were methodologically weak, and only our more rigorous design failed to find effects.”

4. Minor comments

In the Methods section (page 6, line 119), the phrase “contradictions to safe participation” appears to be a typographical error and likely should read “contraindications to safe participation.” I would also recommend a careful proofread of the manuscript to check for and correct any remaining minor wording or spelling issues prior to publication.

[Note: HTML markup is below. Please do not edit.]

Reviewers' comments:

Reviewer's Responses to Questions

**Comments to the Author**

1. Is the manuscript technically sound, and do the data support the conclusions?

Reviewer #1: Yes

Reviewer #2: Yes

**********

2. Has the statistical analysis been performed appropriately and rigorously?

Reviewer #1: Yes

Reviewer #2: Yes

**********

3. Have the authors made all data underlying the findings in their manuscript fully available?

The PLOS Data policy

Reviewer #1: Yes

Reviewer #2: Yes

**********

4. Is the manuscript presented in an intelligible fashion and written in standard English?

Reviewer #1: Yes

Reviewer #2: Yes

**********

Reviewer #1: Here are my comments

1. Abstract: The authors stated that “We predicted that, relative to the PMR active control...” Predicted must be changed to hypothesize.

2. Sample size: There must be a clearly defined section titled sample size. What informed the treatment effect size of partial eta-squared of 0.02? A reference or justification must be provided. What was the power of the study and the margin of error used in estimating the sample size? Did you adjust the sample size for non-response rate?

3. Under study design, provide the name of the specific study design that was used before you continue with the detailed description of the study. Eg, this study employed an RCT design....

4. There must be a section titled outcome measures that describes each key outcome measure (primary and secondary outcomes if applicable) and how it was measured. The information is all lumped together, making it difficult to follow the manuscript. There should be another section titled “Description of the intervention”.

5. Wondering why two different versions of the same statistical software were used in analysing the data. Any explanation

6. What key assumptions were considered for the use of the two-tailed t-test, analyses of variance (ANOVA), and linear mixed models, and how were these assumptions tested to warrant their use? These details must be provided in the manuscript

Reviewer #2: 1. Brief summary

This study is a randomized controlled trial comparing a 4-week online Loving-Kindness Meditation (LKM) program with Progressive Muscle Relaxation (PMR) in healthy adults, evaluating social well-being and experimentally induced acute pain using a wall sit task. Contrary to the hypothesis that LKM would enhance social well-being and thereby improve pain tolerance and reduce subjective pain, no significant between-group differences were observed on pain-related outcomes, and increases in perceived social support and a trend toward reduced loneliness were observed only in the PMR group. In addition, the trial was conducted entirely online during the early phase of the COVID 19 pandemic, which constitutes an important contextual feature of this study.

2. Strengths

- The authors increased the transparency and reproducibility of the study by registering the trial on ClinicalTrials.gov and preregistering the hypotheses, design, and analysis plan on OSF.

- The use of stratified block randomization by sex and age with envelope-based allocation concealment, together with an ITT approach and linear mixed models, represents a relatively rigorous design and analysis for an RCT.

- The LKM and PMR intervention protocols (4 weeks, 6 sessions per week, 15 minutes per session) are well standardized, and adherence is carefully monitored via Qualtrics by tracking session duration and completion status.

- Social well-being and its context are assessed in a multidimensional way (perceived social support, loneliness, anxiety, daily social and non-social activities, and COVID 19–related indicators), providing rich data to interpret null and partially positive findings.

- Finally, the introduction of a wall sit task delivered via videoconference as a remote pain-induction paradigm is a methodological strength, demonstrating that acute pain can be measured in a reasonably reliable way in an online setting.

3. Major comments

1) Abstract

It would be helpful if the abstract explicitly stated that this study uses a randomized controlled trial (RCT) design. If possible, in line with the CONSORT for Abstracts recommendations, I would also suggest adding one brief sentence describing the level of blinding (e.g., whether the trial was open-label) and the basic randomization procedure.

In addition, since the abstract mentions the COVID 19 context, it may help readers interpret the findings if you could add one sentence indicating how this period might plausibly have influenced social support or pain reporting (e.g., by restricting in-person social contact or increasing background stress)

2) Introduction

The Introduction clearly presents the study hypothesis that LKM affects pain via improvements in social well-being. However, adding a conceptual figure at the end of the Introduction that schematically depicts this hypothesized pathway would, in my view, help readers grasp the overall structure of the study and its key variables at a glance.

In addition, while LKM is introduced in the Introduction in relatively considerable detail as an intervention with various positive effects on social, emotional, and pain-related outcomes, PMR is mentioned only briefly, mainly to note that it has been shown to reduce anxiety but has not been reported to enhance social connectedness. It would therefore be helpful to provide a clearer theoretical rationale for why PMR was selected as the active control intervention in this study.

In particular, for readers who may not be familiar with either LKM or PMR, the fact that the pain induction method used in this study is the wall sit test—which imposes a load on the lower body muscles—may intuitively lead them to assume that PMR, which directly targets muscular relaxation, could be more effective in reducing pain. Considering this point, it would be helpful if the Introduction more clearly explained the theoretical reasoning for why PMR was considered an appropriate control condition in this context.

As this is my personal interpretation, clarifying the authors’ intended rationale for this comparison would likely help readers better understand the logic underlying the study.

3) Methods

I consider it a strength that the meditation sessions were delivered via pre-recorded audio through Qualtrics and that session duration and completion were tracked in real time, as this allows for an objective assessment of intervention adherence. However, this approach can only confirm that the audio file was played to the end, and it does not guarantee that participants actually followed the meditation instructions and remained focused on the practice during that time. If possible, in future studies, it may be useful to include brief self-report measures of practice engagement (e.g., perceived immersion, distraction) so that not only the quantity but also the quality of meditation practice can be evaluated.

In the Methods section, the authors describe using the wall sit test as a remotely administered experimental pain task, but the theoretical and practical reasons for choosing the wall sit over other possible pain induction methods are not fully elaborated. Providing a more detailed rationale for this choice would, in my view, help readers better understand and evaluate the suitability of the selected pain paradigm.

4) Discussion

The final sentence of the first paragraph of the Discussion (“there may be more effective methods than meditation”) carries a risk of being interpreted as a general conclusion that meditation as a whole might be less effective than other interventions, beyond the specific context of this trial (healthy adults, a 4 week online intervention, and the early phase of the COVID 19 pandemic). Given that the results of the present study (1) showed no clear effects of either LKM or PMR on pain outcomes, (2) found only partial evidence of increased social support and a trend toward reduced loneliness in the PMR group, and (3) are based on a relatively modest sample size (n = 69), it would seem more appropriate to temper this sentence so that the conclusion is clearly restricted to the particular conditions and protocols examined in this study.

I find it very helpful that the Discussion highlights several methodological limitations of prior LKM studies, such as small sample sizes, weakly designed control conditions, over-reliance on marginally significant findings, and potential experimenter bias when the meditation teacher is a co author. At the same time, the present study also has its own constraints, including a healthy volunteer sample, the use of a single experimental pain paradigm, and the unique circumstances of the COVID 19 pandemic. More explicitly acknowledging these limitations of the current study alongside the critique of the existing LKM literature could help reduce the risk that readers interpret the message as “prior studies were methodologically weak, and only our more rigorous design failed to find effects.”

4. Minor comments

In the Methods section (page 6, line 119), the phrase “contradictions to safe participation” appears to be a typographical error and likely should read “contraindications to safe participation.” I would also recommend a careful proofread of the manuscript to check for and correct any remaining minor wording or spelling issues prior to publication.

**********

what does this mean?). If published, this will include your full peer review and any attached files.

If you choose “no”, your identity will remain anonymous but your review may still be made public.

**Do you want your identity to be public for this peer review?**  For information about this choice, including consent withdrawal, please see our Privacy Policy

Reviewer #1: No

Reviewer #2: No

**********

[NOTE: If reviewer comments were submitted as an attachment file, they will be attached to this email and accessible via the submission site. Please log into your account, locate the manuscript record, and check for the action link "View Attachments". If this link does not appear, there are no attachment files.]

To ensure your figures meet our technical requirements, please review our figure guidelines: https://journals.plos.org/plosone/s/figures

You may also use PLOS’s free figure tool, NAAS, to help you prepare publication quality figures: https://journals.plos.org/plosone/s/figures#loc-tools-for-figure-preparation.

NAAS will assess whether your figures meet our technical requirements by comparing each figure against our figure specifications.

Attachment Submitted filename: Review_Comments_PONE-D-25-57691.docx

# Author comment: Author response to Decision Letter 1

**DOI:** 10.1371/journal.pone.0357888.r002

1 Jun 2026

We thank the Editor and the two reviewers for their thorough and constructive feedback on our manuscript. The comments have improved the clarity and transparency of the manuscript. Below we respond to each comment in turn. Reviewer comments are in italics and our responses follow. All revisions have been implemented using tracked changes in the revised manuscript; section, paragraph, and figure references below refer to the revised version.

Reviewer 1

Comment 1.1. Abstract: The authors stated that “We predicted that, relative to the PMR active control...” Predicted must be changed to hypothesize.

Response: We have changed “predicted” to “hypothesized” in the Abstract and Discussion.

Comment 1.2. Sample size: There must be a clearly defined section titled sample size. What informed the treatment effect size of partial eta-squared of 0.02? A reference or justification must be provided. What was the power of the study and the margin of error used in estimating the sample size? Did you adjust the sample size for non-response rate?

Response: We expanded the Sample size subsection of the Methods to present the a priori power analysis that we pre-registered on the Open Science Framework. Specifically, we now report that the primary power calculation targeted a treatment effect of partial η² = 0.02 on change in pain tolerance in a 2 (meditation group) × 2 (time) repeated-measures design, assuming a correlation between repeated measures of r = .75. This target effect size was selected as a conservative estimate consistent with the modest effects reported in prior research linking social well-being interventions to experimental pain outcomes in healthy adults (e.g., Roberts et al., 2015; Johnson & Dunbar, 2016)

An a priori analysis in G*Power yielded a required total sample of N = 68 (n = 34 per arm) to achieve 90% power at a two-tailed α = .05. With N = 68, we also expected greater than 80% power to detect group differences on self-reported social well-being outcomes given prior effect sizes of Cohen’s d = 0.83–1.45, using a Bonferroni-adjusted α = .05/4 = .0125 to accommodate four planned comparisons. To accommodate an anticipated attrition and non-response rate of approximately 10%, target enrollment was set at 74.

This target effect size was selected as a conservative estimate consistent with the modest effects reported in prior research linking social well-being interventions to experimental pain outcomes in healthy adults (e.g., Roberts et al., 2015; Johnson & Dunbar, 2016)

We did not specify a separate margin of error, but a study powered to detect a minimum effect of partial η² = 0.02 will tend to produce confidence intervals that are reasonably narrow. The achieved N = 69 (75 enrolled, 6 lost to attrition) met the pre-registered target.

Comment 1.3. Under study design, provide the name of the specific study design that was used before you continue with the detailed description of the study.

Response: We now explicitly refer to the design as a “two-arm, parallel-group randomized controlled trial” consistently in the Methods.

Comment 1.4. There must be a section titled outcome measures that describes each key outcome measure… and how it was measured… There should be another section titled“Description of the intervention.”

Response: We added two new subsection headings in the Methods: “Description of the Intervention” (introducing the LKM and PMR content) and “Outcome Measures” (introducing the subjective social well-being measures and pain outcomes). We agree the edits clarify the organization.

Comment 1.5. Wondering why two different versions of the same statistical software were used in analyzing the data.

Response: The initial analyses (conducted at the time of pre-registration and primary analysis) were run in STATA 15, which was the version available to the analytic team at that time. Follow-up analyses and output verification during manuscript revision were conducted in STATA 18 after a scheduled upgrade.

Comment 1.6. What key assumptions were considered for the use of the two-tailed t-test, analyses of variance (ANOVA), and linear mixed models, and how were these assumptions tested to warrant their use? These details must be provided in the manuscript

Response: We added a new paragraph at the end of the Statistical inference subsection describing the assumption checks. In brief: normality within each group was assessed by inspecting histograms, Q–Q-plots and homogeneity of variance was assessed with Levene’s test. For linear mixed models, we examined distributions of residuals for normality, and residuals-versus-fitted plots for homoscedasticity and linearity. Potential outlier observations were checked using standardized residuals and Cook’s distance.

Reviewer 2

Comment 2.1. It would be helpful if the abstract explicitly stated that this study uses a randomized controlled trial (RCT) design. If possible, in line with the CONSORT for Abstracts recommendations, I would also suggest adding one brief sentence describing the level of blinding (e.g., whether the trial was open-label) and the basic randomization procedure.

In addition, since the abstract mentions the COVID 19 context, it may help readers interpret the findings if you could add one sentence indicating how this period might plausibly have influenced social support or pain reporting (e.g., by restricting in-person social contact or increasing background stress)

Response: We have revised the Abstract to (a) state explicitly that this is a “two-arm, parallel-group randomized controlled trial design with stratified block randomization and a double-blind baseline session”, and (b) note that data were collected during the early phase of the COVID-19 pandemic, when social well-being and daily activity were atypical.

Comment 2.2. …adding a conceptual figure at the end of the Introduction that schematically depicts this hypothesized pathway would, in my view, help readers grasp the overall structure of the study… It would therefore be helpful to provide a clearer theoretical rationale for why PMR was selected as the active control intervention in this study…

Response: We added a new conceptual figure (Figure 1) and an accompanying caption summarizing the hypothesized pathway by which LKM is expected to influence acute pain tolerance, pain threshold, and subjective pain through improvements in social well-being (social support, loneliness, prosocial activity and social activity), with PMR serving as an active control matched on format, dose, attention, and relaxation but not hypothesized to operate through a social pathway.

We also added a short paragraph to the Introduction describing the rationale for selecting PMR. PMR is a widely used relaxation protocol that shares with LKM the features of guided audio, structured practice, comparable time commitment, focused attention, and a general relaxation response, but does not have a social or affective focus. These features make PMR a strong active control for isolating the specifically social-affective components of LKM.

Related to these changes, we added a sentence about limitations to the Discussion, “While PMR served as a rigorous active control matched on format and expectancy, its own potential effects on reducing pain during the wall sit is a limitation since it may have obscured LKM’s impacts especially since the study did not include a passive control arm. In other words, the null between-group differences reported here could technically reflect two similarly effective interventions rather than the absence of an effect for either.”

Comment 2.3. …this approach can only confirm that the audio file was played to the end, and it does not guarantee that participants actually followed the meditation instructions and remained focused… the theoretical and practical reasons for choosing the wall sit over other possible pain induction methods are not fully elaborated.

Response: We now explain the rationale for choosing the wall sit test over alternative laboratory pain paradigms (cold pressor, thermal stimulation, pressure algometry), which require specialized equipment and in-person administration and were therefore not feasible given the remote, videoconference-based design and the COVID-19 context. We also note that ischemic, exercise-based tasks such as the wall sit allow for standardized, self-terminated pain induction with minimal equipment and preserve ecological validity, and that pain outcomes in the wall sit have previously been linked to social outcomes. In the newly added Limitations paragraph in the Discussion, we now also acknowledge that participant engagement with session audio was indexed primarily through self-report and that future work should incorporate more objective engagement metric tests.

Comment 2.4. The final sentence of the first paragraph of the Discussion (“there may be more effective methods than meditation”) carries a risk of being interpreted as a general conclusion… it would seem more appropriate to temper this sentence so that the conclusion is clearly restricted to the particular conditions and protocols examined in this study.

Response: We agree and have revised the Discussion accordingly. The revised statement now reads, “In sum, our findings suggest that interventions that improve social support could potentially benefit acute pain tolerance in healthy adults, but do not establish that brief, online LKM is effective for this purpose. Further research directly comparing LKM with other social-support-enhancing interventions could clarify relative efficacy.”

Comment 2.4. (continued) …the present study also has its own constraints, including a healthy volunteer sample, the use of a single experimental pain paradigm, and the unique circumstances of the COVID 19 pandemic. More explicitly acknowledging these limitations of the current study alongside the critique of the existing LKM literature could help reduce the risk that readers interpret the message as “prior studies were methodologically weak, and only our more rigorous design failed to find effects.”

Response: We added the following text to the Discussion, “Although our design addresses some of methodological concerns raised about prior LKM research, it remains limited in other ways. For example, its reliance on a single experimental pain paradigm conducted only in healthy volunteers and the unique circumstances of the COVID-19 pandemic limit generalizability. These and other limitations discussed below suggest that the present null findings should be interpreted as one informative data point within an evolving literature rather than as definitive evidence.”

Comment 2.5. Minor: “contradictions” should read “contraindications”.

Response: Corrected.

Attachment Submitted filename: Online LKM Response to Reviewer Comments.docx

# Peer review: Decision Letter 1

**DOI:** 10.1371/journal.pone.0357888.r003

**Authors:** Nik Hisamuddin Nik Ab. Rahman

24 Aug 2026

Assessing the effect of Loving-Kindness Meditation on pain and social well-being using a randomized controlled trial design

PONE-D-25-57691R1

Dear Dr. O'Connell,

We’re pleased to inform you that your manuscript has been judged scientifically suitable for publication and will be formally accepted for publication once it meets all outstanding technical requirements.

Within one week, you’ll receive an e-mail detailing the required amendments. When these have been addressed, you’ll receive a formal acceptance letter and your manuscript will be scheduled for publication.

An invoice will be generated when your article is formally accepted. Please note, if your institution has a publishing partnership with PLOS and your article meets the relevant criteria, all or part of your publication costs will be covered. Please make sure your user information is up-to-date by logging into Editorial Manager at Editorial Manager® and clicking the ‘Update My Information' link at the top of the page. For questions related to billing, please contact billing support.

If your institution or institutions have a press office, please notify them about your upcoming paper to help maximize its impact. If they’ll be preparing press materials, please inform our press team as soon as possible -- no later than 48 hours after receiving the formal acceptance. Your manuscript will remain under strict press embargo until 2 pm Eastern Time on the date of publication. For more information, please contact onepress@plos.org.

Kind regards,

Nik Hisamuddin Nik Ab. Rahman

Academic Editor

PLOS One

Additional Editor Comments (optional):

Reviewers' comments:

Reviewer's Responses to Questions

**Comments to the Author**

Reviewer #2: All comments have been addressed

Reviewer #3: All comments have been addressed

Reviewer #4: All comments have been addressed

**********

2. Is the manuscript technically sound, and do the data support the conclusions?

Reviewer #2: Yes

Reviewer #3: Partly

Reviewer #4: Yes

**********

3. Has the statistical analysis been performed appropriately and rigorously?

Reviewer #2: Yes

Reviewer #3: Yes

Reviewer #4: I Don't Know

**********

4. Have the authors made all data underlying the findings in their manuscript fully available?

The PLOS Data policy

Reviewer #2: Yes

Reviewer #3: Yes

Reviewer #4: Yes

**********

5. Is the manuscript presented in an intelligible fashion and written in standard English?

Reviewer #2: Yes

Reviewer #3: Yes

Reviewer #4: Yes

**********

Reviewer #2: The authors have carefully addressed the reviewer comments and substantially improved the manuscript. The addition of the conceptual model, clarification of the rationale for the active control condition, and expanded discussion of study limitations have strengthened the overall quality and interpretation of the findings. Although participant engagement with the meditation intervention remains difficult to verify objectively, this issue has now been appropriately acknowledged as a limitation. Overall, I believe the authors have adequately addressed my concerns, and I have no further revision requests.

Reviewer #3: Good changes have been made based on the reviewers' feedback, but a few things need clarification.

The necessity of using LKM and the researcher's motivation for using this method should be further explored.

Your reason for choosing PMR as the comparative intervention needs to be explained.

The explanation about using two softwares for data analysis was not convincing.

Reviewer #4: Dear Authors,

The manuscript addresses an important topic and reflects considerable effort in filling the gap in the existing literature. You have addressed the reviewer comments. The manuscript now looks more comprehensive.

However, Please reform the title to make it more clear; it could be more clear if you include participants in the title: For eg: Effects of Loving-Kindness Meditation on pain and social well-being among:…….a randomized controlled trial design, and organise your writing in method section for more clarity.

All the best!

Mira

**********

what does this mean?). If published, this will include your full peer review and any attached files.

If you choose “no”, your identity will remain anonymous but your review may still be made public.

**Do you want your identity to be public for this peer review?**  For information about this choice, including consent withdrawal, please see our Privacy Policy

Reviewer #2: No

Reviewer #3: No

Reviewer #4: No

**********

# Editor report: Acceptance letter

**DOI:** 10.1371/journal.pone.0357888.r004

**Authors:** Nik Hisamuddin Nik Ab. Rahman

PONE-D-25-57691R1

PLOS One

Dear Dr. O'Connell,

I'm pleased to inform you that your manuscript has been deemed suitable for publication in PLOS One. Congratulations! Your manuscript is now being handed over to our production team.

At this stage, our production department will prepare your paper for publication. This includes ensuring the following:

* All references, tables, and figures are properly cited

* All relevant supporting information is included in the manuscript submission,

* There are no issues that prevent the paper from being properly typeset

You will receive further instructions from the production team, including instructions on how to review your proof when it is ready. Please keep in mind that we are working through a large volume of accepted articles, so please give us a few days to review your paper and let you know the next and final steps.

Lastly, if your institution or institutions have a press office, please let them know about your upcoming paper now to help maximize its impact. If they'll be preparing press materials, please inform our press team within the next 48 hours. Your manuscript will remain under strict press embargo until 2 pm Eastern Time on the date of publication. For more information, please contact onepress@plos.org.

You will receive an invoice from PLOS for your publication fee after your manuscript has reached the completed accept phase. If you receive an email requesting payment before acceptance or for any other service, this may be a phishing scheme. Learn how to identify phishing emails and protect your accounts at https://explore.plos.org/phishing.

If we can help with anything else, please email us at customercare@plos.org.

Thank you for submitting your work to PLOS One and supporting open access.

Kind regards,

PLOS One Editorial Office Staff

on behalf of

Professor Dr Nik Hisamuddin Nik Ab. Rahman

Academic Editor

PLOS One
